# Time dependent asymptotic analysis of the gene regulatory network of the AcrAB-TolC efflux pump system in gram-negative bacteria

**DOI:** 10.1007/s00285-021-01576-4

**Published:** 2021-03-10

**Authors:** George H. Youlden, Vito Ricci, Xuan Wang-Kan, Laura J. V. Piddock, Sara Jabbari, John R. King

**Affiliations:** 1 School of Mathematics, University of Birmingham, Birmingham, B15 2TT UK; 2 School of Mathematical Sciences, University of Nottingham, Nottingham, NG7 2RD UK; 3 Institute of Microbiology and Infection, University of Birmingham, Birmingham, B15 2TT UK; 4 Gyrd-Hansen Group, Ludwig Institute for Cancer Research, Nuffield Department of Medicine, University of Oxford, Oxford, OX3 7DQ UK

**Keywords:** *Salmonella*, *Escherichia coli*, AcrAB-TolC, Mathematical modelling, Asymptotic analysis, 34E13, 92B05, 37N25

## Abstract

Efflux pumps are a mechanism of intrinsic and evolved resistance in bacteria. If an efflux pump can expel an antibiotic so that its concentration within the cell is below a killing threshold the bacteria are resistant to the antibiotic. Efflux pumps may be specific or they may pump various different substances. This is why many efflux pumps confer multi drug resistance (MDR). In particular over expression of the AcrAB−TolC efflux pump system confers MDR in both *Salmonella* and *Escherichia coli*. We consider the complex gene regulation network that controls expression of genes central to controlling the efflux associated genes *acrAB* and *acrEF* in *Salmonella*. We present the first mathematical model of this gene regulatory network in the form of a system of ordinary differential equations. Using a time dependent asymptotic analysis, we examine in detail the behaviour of the efflux system on various different timescales. Asymptotic approximations of the steady states provide an analytical comparison of targets for efflux inhibition.

## Introduction

### Antimicrobial resistance

Antibiotics are used to treat bacterial infections, by interfering with the growth or other essential mechanisms for survival of the bacteria. These essential mechanisms can include maintaining the structure of the cell envelope, protein production and DNA replication (Sköld 2011). The use of antibiotics has been prevalent since the introduction of sulphonamides, the first antibiotics used in clinics, in the early 1930s (Miller and Bohnhoff 1950). However, the widespread use of antibiotics has exerted selection pressures on bacteria, causing mutant antibiotic resistant strains to evolve. There are currently 17 classes of antibiotics, but for each of these a mechanism for resistance has emerged (Davies and Davies 2010). Whilst the development of new antibiotics is a possibility for treating these resistant bacteria, the discovery of new antibiotics has slowed within the twenty first century, with the possibility of a post antibiotic era in the coming years (Alanis 2005). Thus, there is a huge need to look into alternative and novel treatments to treat bacterial infections.

In February 2017, the World Health Organisation (WHO) released a priority list of antibiotic resistant bacteria in need of treatment strategies. *Enterobacterales* resistant to carbapenem and cephalosporin were classified as critical for the development of new antibiotics. This is a large group of Gram-negative bacteria, which includes *Salmonella* spp. and *Escherichia coli.* Additionally, fluoroquinolone-resistant *Salmonella* spp. was deemed high priority. *Salmonella* spp. is a genus of rod shaped Gram-negative pathogenic bacteria that is one of the main causes of intestinal infections from food, most commonly from poultry products. In most cases of infection, antibiotics are not needed, however for salmonellosis in immunosuppressed patients, invasive non-typhoidal *Salmonella* infections (iNTS) and S. Typhi antibiotics are necessary (Feasey et al. 2012; Rowe et al. 1997). S. Typhi, which causes Typhoid fever, can transmit from human to human by the fecal to oral route, and hence bad sanitation is a leading cause of transmission (Ryan et al. 2004). Multi drug resistant (MDR) strains of S. Typhi have developed, most prominently in South Asia and Africa. High mortality rates are highly prominent in developing countries in these regions, due to poor sanitation and the high prevalence of immunodeficiency diseases (Rowe et al. 1997).

### Efflux pumps

Efflux pumps are a mechanism of intrinsic and evolved resistance in bacteria. They are transport proteins found in the cell membrane that expel substances into the external surrounding environment. If an efflux pump can expel an antibiotic so that its concentration within the cell is below a killing threshold the bacteria are resistant to the antibiotic. Efflux pumps can be specific or they may pump various different substances and compounds and confer MDR. Many MDR bacteria exhibit over-expression of efflux pumps (Blair et al. 2014b). Over-expression is often caused by mutations in local gene repressors and changes to transcriptional regulators that affect the production of proteins associated with efflux (Webber and Piddock 2003).

**Fig. 1:**
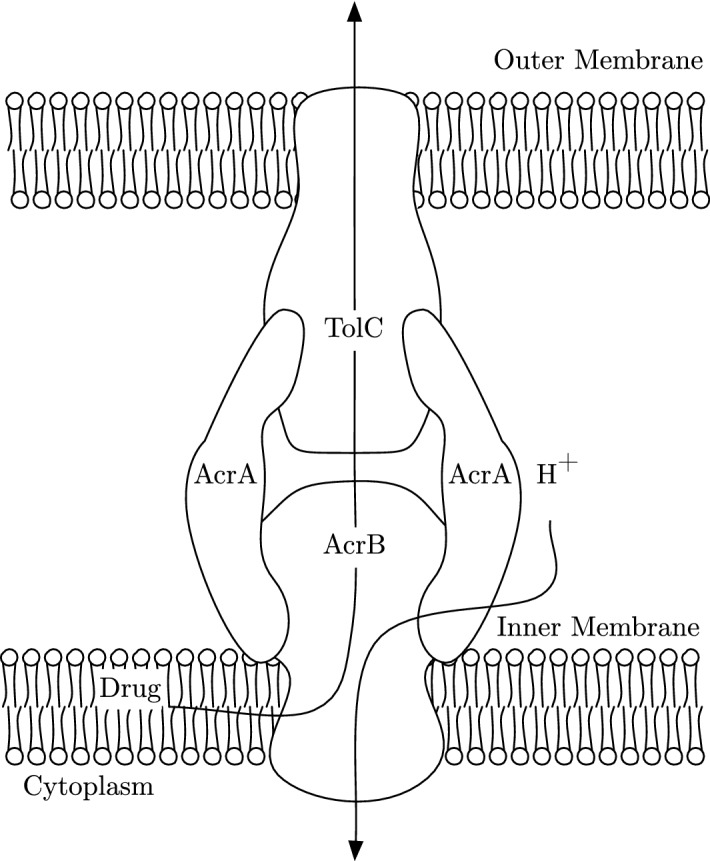
The AcrAB-TolC system common in *E. coli* and *Salmonella*. Drug is exported from within the cell via proton motive force, powered by hydrogen ions / protons $(H^{+})$, travelling into the cell along its concentration gradient

Many efflux pumps that exhibit MDR are part of the Resistance Nodulation Division family (RND) and made of three proteins together spanning the inner and outer membranes, meaning they can expel a substance from the cell to the outside. In particular, a member of this family is the AcrAB-TolC system which is common in *Enterobacterales*. We exhibit this system in Fig. 1. The AcrAB-TolC system is tripartite, composing of the transporter protein AcrB, the periplasmic adaptor protein AcrA and an outer membrane protein TolC. The energy to efflux drugs and other substances through the AcrAB-TolC system is provided by proton motive force (PMF) that moves hydrogen ions from the bacterial periplasm to the cytoplasm. This movement causes an electrochemical gradient that drives transport of the drug through the efflux pump, expelling the drug or other toxic substances from the bacteria (Piddock 2006).

### Gene regulatory networks

**Fig. 2:**
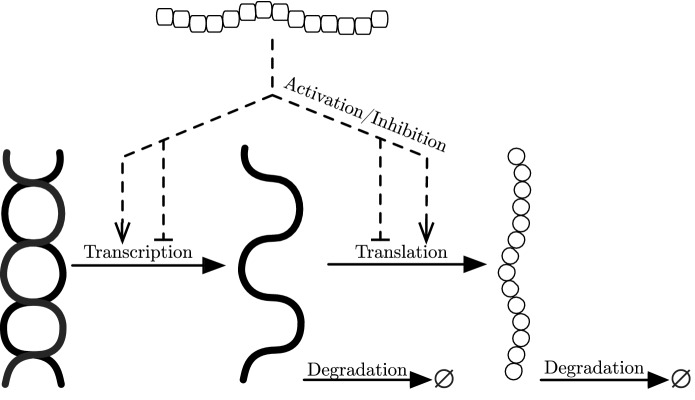
Processes of gene regulation and protein synthesis. We exhibit here a gene in the DNA (double helix) being transcribed to mRNA (single strand); the mRNA is then translated to create a protein (circle chain). Here both mRNA and protein undergo degradation. We also exhibit by the dashed lines the potential activation or inhibition from a protein within the system (rounded square chain) on the transcription and translation processes. We note that we only display here pre translational regulation. However, post translational regulation acting upon proteins, for example breaking down of proteins via enzymes also occurs

In response to environmental stimuli, bacteria are able to control expression of certain genes via gene regulatory networks (GRNs). This includes altering the expression of efflux pump genes in response to an antibiotic or other substance toxic to bacteria. When a gene is expressed, the processes of transcription (mRNA synthesis from a DNA template) and translation (protein synthesis from mRNA by ribosomes) occur. In bacteria, translation in most cases takes place as soon as transcription of mRNA occurs. This is due to the lack of a nuclear membrane in bacteria and the high instability and degradation of mRNA molecules. In addition, one strand of mRNA can be translated multiple times before it is degraded. For these reasons, bacteria can quickly adapt to changes in environmental stimuli. Certain genes, however, are not expressed constitutively as they may be part of a regulatory network that controls activation and/or repression of the gene’s transcription via regulatory proteins. We exhibit these processes in Fig. 2.

All bacteria have complicated networks of genes controlling gene expression, which enables them to change their behaviour depending on which genes are expressed and in what quantities (Wilson et al. 2002). Gram-negative bacteria express several efflux pumps in their cell membranes. The AcrAB-TolC pump provides intrinsic resistance to various antibiotics. Overexpression of this efflux pump confers MDR. However, regulation of efflux pump expression is complicated and it is, therefore, important to understand the processes governing it. We use mathematical modelling techniques to represent the gene regulatory network governing AcrAB expression with a system of ordinary differential equations (ODEs). This will further our understanding of the regulation of these genes and hence the AcrAB-TolC system, enabling us to look into potential mechanisms to inhibit efflux and hence counter MDR.

### Mathematical models of efflux pumps and gene regulatory networks

While mathematical models of efflux pump mechanisms exist, to our knowledge there are no published mathematical models of the GRNs governing efflux regulation in bacteria.

In terms of general efflux pump models, Nagano and Nikaido (2009) present a model of antibiotic efflux in *E. coli*, based on the AcrAB-TolC efflux pump system. This model includes the enzyme $\beta$-lactamase located in the periplasm, which breaks down $\beta$-lactam antibiotics such as penicillin. They assume substances expelled by efflux undergo Michaelis-Menten kinetics, with diffusion into the cell given by Fick’s law. By using parameter fitting techniques, they are able to estimate various binding coefficients for certain antibiotics with AcrB. Lim and Nikaido (2010) continue this work, extending the study to find binding coefficients for various different penicillins.

The AcrAB-TolC efflux pump system and genes that govern the system’s expression have been the topic of other mathematical models. Rossi et al. (2018) experimentally manipulated the degradation of MarA (a known activator of *acrAB* expression) in *E. coli*, to see the resulting effects on downstream genes. A generic mathematical model was formulated consisting of three genes: an activator and two downstream genes. This model showed that activators with a long half life had an advantage by increasing the coordination of the downstream genes. The analytical results were replicated experimentally with *marA* and downstream genes *inaA* and *acrAB*. Langevin and Dunlop (2018) produced a mathematical model governing the stress tolerance of *E. coli* and the cost of expressing the AcrAB-TolC efflux pump system. They competed *acrB* knockout strains against strains with active *acrB* expression, measuring the population size of each strain over time with different environmental stress conditions. A mathematical model on the biomass and substrate availability was formulated that displayed a strong alignment to experimental data. Both of these models give great insights into the expression of selected genes. In this study, we incorporate the interplay of the larger network that governs *acrAB* expression.

Efflux pumps are also modelled in eukaryotic cells, for example efflux pumps associated with MDR in cancer patients. Michelson and Slate (Michelson and Slate 1992, 1994) present a model of the p-glycoprotein pump. This pump is energy dependent, meaning it relies on the process of dephosphorylation of adenosine triphosphate (ATP) to adenosine diphosphate (ADP) to function. After modelling the transport of drug through this efflux pump, they included the presence of an inhibitor that prevents the drug from binding to the pump, modelling situations of competitive inhibition, and non-competitive inhibition. Yi et al. (1999) develop a single cell model that encompasses drug delivery and efflux simultaneously to look into MDR of cancer cells. In this model efflux is modelled through active transport using Michaelis-Menten equations, building from Michelson and Slate. Diao et al. (2016) produce a model of a yeast efflux pump found in *Saccharomyces cerevisiae*. They model the negative feedback loop of a regulator, efflux pump and inducer (a substrate of the efflux pump). Charlebois et al. (2014) also produce a model of the efflux pump in *Saccharomyces cerevisiae*. Here a more complex model is produced, consisting of three genes that are part of a drug resistance network involved with efflux pump expression.

There are various methods used to model GRNs, see (Karlebach and Shamir 2008) for a review. Glass and Kauffman (1973) were the first to present a Boolean model of GRNs. Here, they propose modelling genes as switches of expression where they can be active (1) or inactive (0). Weaver et al. (1999) present a linear model of GRNs, in which each gene’s expression level depends on a summation of the levels of its regulators. Nachman et al. (2004) increase the level of detail in their model of GRNs by delving deeper into transcription, including transcriptional factors that could bind to the promoter site of a gene. More closely related to this study, in Jabbari et al. (2010) and Jabbari et al. (2015), models of the GRNs that govern quorum sensing in *Staphylococcus aureus* and toxin production in *C. difficile* are presented. In these studies, due to a lack of available data for parameterisation, the models are nondimensionalised and time-dependent asymptotic analyses are performed using the relative sizes of the nondimensional parameters. The analyses provide insights into the behaviours of the systems on various different timescales. By modelling the GRN behind efflux pump expression, we can gain insight into the most influential aspects of the network. After formulating our model, we will perform an asymptotic analysis. From this, we consider various targets for inhibiting efflux pump expression and hence combating MDR.

## Model formulation

### Dimensional model

**Fig. 3:**
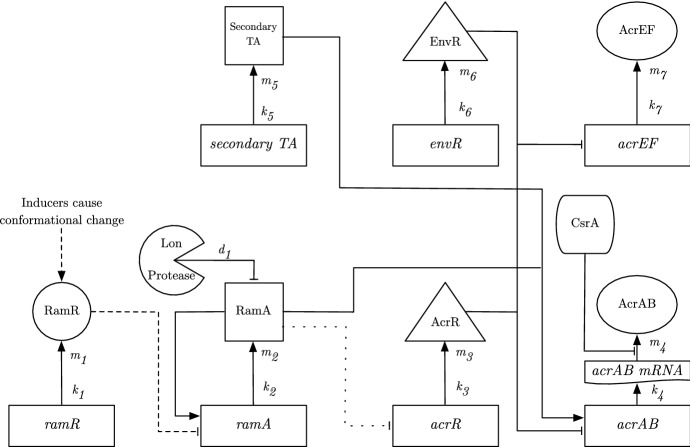
A schematic diagram exhibiting the regulation of acrAB expression.In the rectangles we have the genes involved in this network, the shapes first linked out from these genes are the proteins produced by them (we omit most mRNA stages for simplicity). The two shapes not linked to the genes are the enzyme Lon Protease and the translation activator CsrA. Solid lines capture the behaviour of both the wild-type and RamR variant, while the dashed line is relevant only for the wild-type. The dotted line shows potential inhibition of a*acrR* expression

To formulate our model of the GRN governing *acrAB* expression, we must first delve into the processes governing the GRN. We exhibit a detailed schematic of the GRN in Fig. 3. We note that in this network, we also include the homologue of *acrAB*, *acrEF*. Experimentally, this efflux pump gene expression has been shown to become more prevalent when there is less production of AcrAB or the *acrAB* genes are deleted, inactivated, or when the protein is produced, but nonfuctional (Wang-Kan et al. 2017).

To consider as much of the regulatory network around efflux as possible, we assume that the cells are subject to stress, e.g. antibiotic or oxidative stress. This enables us to bring in the transcriptional activators (TAs) RamA, SoxS, MarA and/or Rob, which can all regulate the expression of *acrAB* and *tolC* genes. Each of these activators is produced in response to a specific stress. Under stress, de-repression of the relevant TA occurs and the TA binds to a shared binding site on the promoter region of *acrAB*, activating its expression. Post transcription of *acrAB*, the RNA binding protein CsrA acts as a stabiliser of translation of *acrAB* mRNA into AcrAB protein. *acrAB* expression is also inhibited by the local repressor protein AcrR, whilst the homologue efflux pump *acrEF* expression is inhibited by its own local repressor protein EnvR (also called AcrS). We note that the protein EnvR is also capable of inhibiting *acrAB* expression at the same binding site as AcrR.

Of the TAs introduced above, RamA is the primary TA of efflux. RamA is itself subject to regulation: it is degraded post-translationally by the enzyme Lon protease to ensure its levels are returned to basal levels in the absence of stress (Ricci et al. 2014), and transcription of *ramA* is subject to repression by RamR and positive autoregulation by RamA itself. We also consider a specific strain of S. Typhimurium (SL1344) that displays MDR as a consequence of a *ramR::aph* mutation in the *ramR* gene, resulting in production of a non-functional RamR protein that is unable to repress *ramA* expression, thus indirectly causing over-expression of *acrAB* genes and multi drug resistance.

Mathematically, SoxS, MarA and/or Rob would have equivalent representations, each triggered by a different stress. For simplicity, therefore, we only include one variable to represent these and refer to it as the secondary TA. The genes that govern expression of these proteins also have their own repressors that are involved in the network (for example SoxR, and MarR), however as we are assuming that these genes are expressed constitutively (when the bacteria are under stress), we do not include the repressor genes in our model for simplicity.

It has been shown experimentally that with decreased *acrAB* expression, greater expression of the homologue efflux pump gene *acrEF* occurs (Blair et al. 2014a). This may be through known mechanisms in the network, for example through the repressor *envR*. However, since these mechanisms have not yet been fully elucidated, we capture this behaviour in the model by a simple direct link between AcrAB levels and expression of *acrEF*, noting that this is an area where extra detail can be incorporated in future work. We also note that RamA protein may bind at the *acrR* gene which could potentially disrupt the levels of AcrR, either by direct inhibition of transcription, or by preventing activators from binding.

We note here that antibiotic concentration itself could have an effect on the GRN: one would expect that the presence of antibiotic would trigger upregulation of efflux activator and/or downregulation of efflux repressors. However, we here consider the dynamics of the GRN in isolation to focus on the interplay of genes and proteins within the network when subject to a constant stress. These additional feedback effects are the subject of future work.

The equations resulting from these processes are

1 $$\begin{aligned} \frac{dR_m}{dt}&= k_1-\delta _m R_m, \end{aligned}$$

2 $$\begin{aligned} \frac{dA_m}{dt}&= k_2 \frac{K_R A}{(A+K_{A_1})(R+K_R)} +k_2'-\delta _m A_m, \end{aligned}$$

3 $$\begin{aligned} \frac{dC_m}{dt}&=k_3 \frac{K_{A_2}}{A+K_{A_2}}-\delta _m C_m, \end{aligned}$$

4 $$\begin{aligned} \frac{dB_m}{dt}&=k_4 \frac{K_{E_2}K_C(K_S A+ K_{A_1} S)}{(K_C E+K_{E_2}K_C+K_{E_2}C)(K_{A_1}K_S+K_{A_1}S+K_S A)} -\delta _m B_m, \end{aligned}$$

5 $$\begin{aligned} \frac{dS_m}{dt}&=k_5 -\delta _m S_m, \end{aligned}$$

6 $$\begin{aligned} \frac{dE_m}{dt}&=k_6 -\delta _m E_m, \end{aligned}$$

7 $$\begin{aligned} \frac{dF_m}{dt}&=k_7 \frac{K_{E_1}}{K_{E_1}+E} -\delta _m F_m, \end{aligned}$$

8 $$\begin{aligned} \frac{dR}{dt}&= \mu (m_1 R_m - \delta _p R), \end{aligned}$$

9 $$\begin{aligned} \frac{dA}{dt}&= m_2 A_m-\delta _p A -\delta _L A, \end{aligned}$$

10 $$\begin{aligned} \frac{dC}{dt}&=m_3 C_m-\delta _p C, \end{aligned}$$

11 $$\begin{aligned} \frac{dB}{dt}&=m_4 B_m\frac{T_C}{T_C+K_{T_C}}-\delta _p B, \end{aligned}$$

12 $$\begin{aligned} \frac{dS}{dt}&=m_5 S_m-\delta _p S, \end{aligned}$$

13 $$\begin{aligned} \frac{dE}{dt}&=m_6 E_m-\delta _p E, \end{aligned}$$

14 $$\begin{aligned} \frac{dF}{dt}&=m_7 F_m \frac{K_B}{B+K_B}-\delta _p F. \end{aligned}$$

We note that all of the differential equations have linear terms regarding degradation, which we group as the same rate for mRNAs and proteins respectively ($\delta _m$ and $\delta _p$). RamA, SoxS and MarA are the only proteins that we know of that undergo enzyme degradation in the GRN, all degraded by Lon Protease. The secondary TA Rob however does not experience this degradation (Duval and Lister 2013). Since we have grouped together SoxS, MarA and Rob together as secondary TAs, we opt not to include this additional enzyme degradation, instead only including enzyme degradation for RamA in Eq. (9). In the case of transcription and translation however, the terms are not so straightforward. In Eqs. (3), (7), (11) and (14), we have one protein either activating or repressing transcription or translation. The nonlinear term in Eq. (2), reflects two proteins binding at different sites (one activating and one repressing transcription). Furthermore, Eq. (4) reflects two proteins competing for one site (activating transcription) activating transcription and two proteins competing for a different site (repressing transcription). In Eq. (2), we also include a lower basal rate of transcription of *ramA* mRNA. We include this to prevent low or zero transcription of mRNA when there is little RamA protein in the system. Finally, we incorporate the RamR variant via a mutant coefficient ($\mu$) in Eq. (8). By setting this value to zero, we can replicate the case of mutated RamR as this results in no translation of *ramR* mRNA. Conversely by setting this value to one we have full translation, but also degradation of RamR protein.

We denote the variables and parameters used in this model in Tables 1 and 2 respectively. Due to a lack of relevant experimental data, it is not currently possible to estimate absolute parameter values. Instead, we nondimensionalise the model and exploit our biological insight to estimate the relative nondimensional parameter values. In doing so, we can use an asymptotic analysis to compare the wild-type and mutant cases to provide useful insights to counter efflux overexpression and hence MDR in *Salmonella*.

**Table 1:** Variables used in our ODE models along with their respective units

Variables	Concentration of	Units
$R_m$	*ramR* mRNA	nM
*R*	RamR	nM
$A_m$	*ramA* mRNA	nM
*A*	RamA	nM
$C_m$	*acrR* mRNA	nM
*C*	AcrR	nM
$B_m$	*acrAB* mRNA	nM
*B*	AcrAB	nM
$S_m$	secondary TA mRNA	nM
*S*	Secondary TA	nM
$E_m$	*envR* mRNA	nM
*E*	EnvR	nM
$F_m$	*acrEF* mRNA	nM
*F*	AcrEF	nM

**Table 2:** A table of parameters used in our model and their respective units

Parameter	Description	Units
$k_1$	Transcription rate of *ramR* mRNA	nM$s^{-1}$
$m_1$	Translation rate of RamR	$s^{-1}$
$k_2$	Transcription rate of *ramA* mRNA	nM$s^{-1}$
$m_2$	Translation rate of RamA	$s^{-1}$
$k_3$	Transcription rate of *acrR* mRNA	nM$s^{-1}$
$m_3$	Translation rate of AcrR	$s^{-1}$
$k_4$	Transcription rate of *acrAB* mRNA	nM$s^{-1}$
$m_4$	Translation rate of AcrAB	$s^{-1}$
$k_5$	Transcription rate of secondary TA mRNA	nM$s^{-1}$
$m_{5}$	Translation rate of secondary TA	$s^{-1}$
$k_{6}$	Transcription rate of *envR* mRNA	nM$s^{-1}$
$m_{6}$	Translation rate of EnvR	$s^{-1}$
$k_{7}$	Transcription rate of *acrEF* mRNA	nM$s^{-1}$
$m_{7}$	Translation rate of AcrEF	$s^{-1}$
$k_2'$	Lower transcriptional rate of RamA	nM$s^{-1}$
$\delta _m$	Degradation rate of mRNA	$s^{-1}$
$\delta _p$	Degradation rate of proteins	$s^{-1}$
$\delta _L$	Degradation caused by Lon Protease	$s^{-1}$
$K_R$	Dissociation constant of RamR	nM
$K_{A_1}$	Dissociation constant of RamA with *ramA* and *acrAB*	nM
$K_{A_2}$	Dissociation constant of RamA with *acrR*	nM
$K_C$	Dissociation constant of AcrR	nM
$K_{E_1}$	Dissociation constant of EnvR with *acrEF*	nM
$K_{E_2}$	Dissociation constant of EnvR with *acrAB*	nM
$K_S$	Dissociation constant of secondary TA	nM
$K_{T_C}$	Dissociation constant of CsrA	nM
$K_{B}$	Chemical signals constant of AcrAB	nM
$T_C$	CsrA	nM
$\mu$	Mutation coefficient	N/A

### Nondimensional model

We nondimensionalise the model using the following variable scalings

15 $$\begin{aligned} R_m=&\frac{k_1}{\delta _m}R_m^*,&S_m=&\frac{k_5}{\delta _m}S_m^*,&A=&K_AA^*,&E=&K_{E_2}E^*, \nonumber \\ A_m=&\frac{k_2}{\delta _m}A_m^*,&E_m=&\frac{k_6}{\delta _m}E_m^*,&C=&K_CC^*,&F=&\frac{k_7m_7}{\delta _m^2}F^*, \nonumber \\ C_m=&\frac{k_3}{\delta _m}C_m^*,&F_m=&\frac{k_7}{\delta _m}F_m^*,&B=&\frac{k_4}{\delta _m}B^*,&t=&\frac{1}{\delta _m} T, \nonumber \\ B_m=&\frac{k_4}{\delta _m}B_m^*,&R=&K_RR^*,&S=&K_SS^*, \end{aligned}$$

here the asterisks denote nondimensional variables. We have chosen these scalings in order to simplify our system of equations and create nondimensional parameters over which we have insight into their relative sizes. We note that these have the added effect of simplifying the somewhat complex transcription and translation terms. The nondimensionalised parameter groupings that then emerge are as follows

$$\begin{aligned} \varDelta =&\frac{\delta _p}{\delta _m},&\theta =&\frac{k_2 m_2}{\delta _m^2 K_A},&\sigma =&\frac{k_5 m_5}{\delta _m^2 K_S},&\beta =&\frac{m_4 T_C}{\delta _m \left( T_C+K_{T_C}\right) }, \\ \rho =&\frac{k_1 m_1}{\delta _m^2 K_R},&\upsilon =&\frac{\delta _L}{\delta _m},&\eta =&\frac{K_{E_1}}{K_{E_2}},&\xi =&\frac{k_6 m_6}{\delta _m^2 K_{E_2}}, \\ \alpha =&\frac{k_2'}{k_2},&\gamma =&\frac{k_3 m_3}{\delta _m^2 K_C},&\lambda =&\frac{K_{A_1}}{K_{A_2}},&\omega =&\frac{k_4}{\delta _m K_B}. \end{aligned}$$

We assume that all mRNAs and proteins are initially present at a low concentration to monitor how the system upregulates. Thus, we choose low value generic dimensionless initial conditions as follows

16 $$\begin{aligned} R_m^*(0)&=A_m^*(0)=C_m^*(0)=B_m^*(0)=S_m^*(0)=E_m^*(0)=F_m^*(0)=0.01, \nonumber \\ R^*(0)&=A^*(0)=C^*(0)=B^*(0)=S^*(0)=E^*(0)=F^*(0)=0.01. \end{aligned}$$

From here on we will refer to these initial conditions with the following notation. For any gene *X*, we will refer to the mRNA initial condition as $X_{m0}$ and the protein initial condition as $X_{0}$.

### Parameter grouping sizes

By using information about the size of certain parameters compared to others, we can estimate relative parameter sizes within the nondimensional groupings. We start by choosing a parameter grouping that we know to be small (and denote it having the value $\epsilon$):

17 $$\begin{aligned} \alpha =\frac{k_2'}{k_2}=\epsilon . \end{aligned}$$

The grouping $\alpha$ is the ratio of a low basal rate of transcription to the higher transcription rate of *ramA* mRNA. We now assign each other parameter grouping an order of magnitude relative to (17). We assume that

18 $$\begin{aligned} \varDelta =\frac{\delta _p}{\delta _m}=O(\epsilon ), \end{aligned}$$

i.e. mRNA degradation occurs at a much faster rate than the degradation of proteins. We do not know all of the exact degradation rates for the mRNAs and proteins of genes within the network. However, in a similar gram-negative bacteria *E. coli* it was observed that 80% of 4,288 mRNAs had half-lives between 3 and 8 min (Bernstein et al. 2002), whereas for proteins, the vast majority have half-lives of between 5 and 20 hours (Maurizi 1992). In *Salmonella*, on a study of 870 proteins, the calculated median half-life was 99.30 min (Wang et al. 2016). For individual proteins within the GRN, it has been shown for RamA that in a mutant strain with no Lon Protease, there was very little observable degradation within 10 min, indicating that the protein is highly stable (Ricci et al. 2014). Finally, in *E. coli* it has also been observed that AcrA and AcrB lasted for approximately six days (Chai et al. 2016).

At $O(\epsilon ^\frac{1}{2})$ we have the following parameter groupings

19 $$\begin{aligned} \upsilon =&\frac{\delta _L}{\delta _m},&\sigma =&\frac{k_5 m_5}{\delta _m^2 K_S},&\xi =&\frac{k_6 m_6}{\delta _m^2 K_{E_2}}. \end{aligned}$$

Having $\upsilon =O(\epsilon ^\frac{1}{2})$ follows from (18), as the rate of degradation of RamA by Lon protease ($\delta _L$) is larger than the natural rate of protein degradation ($\delta _p$) (Ricci et al. 2014). Thus, we expect this grouping to be a larger order of magnitude than $\varDelta$. For $\sigma$, as the secondary TAs are all underlying activators, we expect that the dissociation constant is relatively large, (furthermore by setting this grouping to this size we obtain the most realistic behaviour). Finally, for $\xi$ the transcription and translation rates for EnvR should be small as this is a repressor of the homologue efflux pump system AcrEF, and thus we expect this grouping to be the same size as $\sigma$ which governs similar underlying genes.

Finally, we have the parameter groupings that we choose to be *O*(1). Firstly,

20 $$\begin{aligned} \lambda =&\frac{K_{A_1}}{K_{A_2}},&\eta =&\frac{K_{E_1}}{K_{E_2}},&\omega =&\frac{k_4}{\delta _m K_B}. \end{aligned}$$

For $\lambda$, the dissociation constants that make up this grouping correspond to the same proteins, but binding to different binding sites. With no evidence to the contrary, we make the assumption that the constants are roughly equal. For $\eta$, there is contradictory evidence in the literature over whether EnvR preferentially binds *acrAB*, *acrEF* or both equally (Hirakawa et al. 2008; Hay et al. 2017). As a result, we also assume these dissociation constants are roughly equal and explore variations to this choice in the parameter sensitivity section. As for $\omega$, we know very little about the chemical signals that cause activation of AcrEF, and hence we keep this as *O*(1) for simplicity. The rest of the *O*(1) parameter groupings are as follows

21 $$\begin{aligned} \theta =&\frac{k_2 m_2}{\delta _m^2 K_A},&\rho =&\frac{k_1 m_1}{\delta _m^2 K_R},&\gamma =&\frac{k_3 m_3}{\delta _m^2 K_C},&\beta =&\frac{m_4 T_C}{\delta _m \left( T_C+K_{T_C}\right) }. \end{aligned}$$

The groupings in (21) correspond to the expression of RamA, RamR, AcrR and AcrAB respectively. These four proteins constitute the primary TAs and the central pathway for the GRN, and thus it is not unreasonable to assume that expression of their genes is relatively high and the respective dissociation constants are likely to be smaller. It has also been shown experimentally in a wild-type *Salmonella* strain, that expression of *ramA* and *acrAB* was higher than *soxS, marA* and *acrEF* (Whitehead et al. 2011). Therefore, we expect these groupings to be the largest in order. Testing more subtle differences in size did not bring significant variations to the behaviour of the model. In Table 3, we summarise all of the above parameter grouping sizes. The parameters below are therefore scaled as follows

**Table 3:** Nondimensionalised parameter groupings and their orders of magnitude

Nondimensional parameter	Size
$\alpha$, $\varDelta$	$O(\epsilon )$
$\upsilon$, $\sigma$, $\xi$	$O(\epsilon ^\frac{1}{2})$
$\rho$, $\theta$, $\gamma$, $\beta$, $\eta$, $\lambda$, $\omega$	*O*(1)

22 $$\begin{aligned} \varDelta =&\epsilon \varDelta ',&\sigma =&\epsilon ^{\frac{1}{2}}\sigma ',&\mu =&\epsilon ^{\frac{1}{2}}\mu ',&\alpha =&\epsilon \alpha ',&\xi =&\epsilon ^{1/2}\xi ', \end{aligned}$$

where the parameters with primes are taken to be *O*(1). Substituting these into our nondimensional model, dropping primes and asterisks, we obtain the following system of equations, where all parameters are *O*(1):

23 $$\begin{aligned} {\frac{{ dR}_{{m}}}{{ dT}}}&=1-R_{{m}}, \end{aligned}$$

24 $$\begin{aligned} {\frac{{ dA}_{{m}}}{{ dT}}}&={\frac{A}{ \left( A+1 \right) \left( R+1 \right) }}+\epsilon \alpha -A_{{m}}, \end{aligned}$$

25 $$\begin{aligned} {\frac{{ dC}_{{m}}}{{ dT}}}&=\frac{\lambda }{A+\lambda }-C_{{m}}, \end{aligned}$$

26 $$\begin{aligned} {\frac{{ dB}_{{m}}}{{ dT}}}&={\frac{A+S}{ \left( 1+S+A \right) \left( 1+E+C \right) }}-B_{{m}}, \end{aligned}$$

27 $$\begin{aligned} {\frac{{ dS}_{{m}}}{{ dT}}}&=1-S_{{m}}, \end{aligned}$$

28 $$\begin{aligned} {\frac{{ dE}_{{m}}}{{ dT}}}&=1-E_{{m}}, \end{aligned}$$

29 $$\begin{aligned} {\frac{{ dF}_{{m}}}{{ dT}}}&={\frac{{ \eta }}{{ \eta }+E}}-F_{{m}}, \end{aligned}$$

30 $$\begin{aligned} {\frac{{ dR}}{{ dT}}}&=\mu \rho \,R_{{m}}-\mu \epsilon \varDelta \,R, \end{aligned}$$

31 $$\begin{aligned} {\frac{{ dA}}{{ dT}}}&=\theta \,A_{{m}}-\epsilon ^{1/2}\upsilon \,A-\epsilon \varDelta \,A, \end{aligned}$$

32 $$\begin{aligned} {\frac{{ dC}}{{ dT}}}&=\gamma \,C_{{m}}-\epsilon \varDelta \,C, \end{aligned}$$

33 $$\begin{aligned} {\frac{{ dB}}{{ dT}}}&=\beta \,B_{{m}}-\epsilon \varDelta \,B, \end{aligned}$$

34 $$\begin{aligned} {\frac{{ dS}}{{ dT}}}&=\epsilon ^{1/2}\sigma \,S_{{m}}-\epsilon \varDelta \,S, \end{aligned}$$

35 $$\begin{aligned} {\frac{{ dE}}{{ dT}}}&=\epsilon ^{1/2}\xi \,E_{{m}}-\epsilon \varDelta \,E, \end{aligned}$$

36 $$\begin{aligned} {\frac{{ dF}}{{ dT}}}&={\frac{F_{{m}}}{\omega B+1}}-\epsilon \varDelta \,F, \end{aligned}$$

with initial conditions (16). We will follow some numerical simulations of the model with a time-dependent asymptotic analysis in order to extract the dominant behaviours over time. Throughout our simulations, we take $\epsilon =0.01$, and all other parameters as unity.

**Fig. 4:**
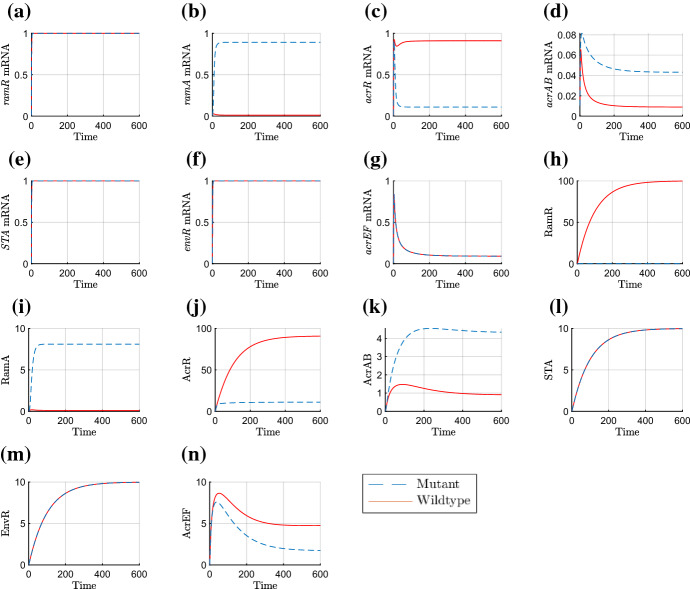
Numerical simulations of the nondimensionalised model (23)–(36) with down regulated initial conditions of 0.01 for all variables. We use $\epsilon =0.01$ and all other parameters are unity. Here STA refers to the secondary TA

## Numerical simulation

We exhibit a numerical simulation of (16) and (23)–(36) in Fig. 4 showing both the wild-type and RamR variant cases. For both cases, we see the rapid production of mRNA (a)-(g), reaching steady state very quickly for most variables. The efflux genes’ mRNAs (d) and (g) reach steady state more slowly due to being affected by regulatory protein concentrations. All proteins (h)-(n) reach steady state at a later timescale than the mRNA. These simulations enable us to exhibit the vast differences between mutant and wild-type strains, caused by the mutation to RamR protein (h). This mutation causes overexpression of *ramA* mRNA (b) and protein (i) which in turn causes lower concentrations of *acrR* mRNA (c) and protein (j). These concentrations combined result in a higher concentration of *acrAB* mRNA (d) and protein (k), which itself causes lower expression of AcrEF (n). We note that the steady state concentration of AcrAB is significantly higher in the mutant case than the wild-type case.

**Table 4:** The scalings for each variable required on each timescale for the wild-type asymptotic analysis. We have $\phi =\ln (1/\epsilon )^{-1}$ and $\delta =\ln (\ln (1/\epsilon ))^{-1}$ which are explained in the timescales in which they feature. We note that production of most mRNAs dominates on the earlier timescales with protein production dominating the later timescales

Timescale	Variable														
	*T*	$R_m$	*R*	$A_m$	*A*	$C_m$	*C*	$B_m$	*B*	$S_m$	*S*	$E_m$	*E*	$F_m$	*F*
1	$\epsilon {\hat{T}}$	$\epsilon {\hat{R}}_m$	$\epsilon {\hat{R}}$	$\epsilon {\hat{A}}_m$	$\epsilon {\hat{A}}$	$\epsilon {\hat{C}}_m$	$\epsilon {\hat{C}}$	$\epsilon {\hat{B}}_m$	$\epsilon {\hat{B}}$	$\epsilon {\hat{S}}_m$	$\epsilon {\hat{S}}$	$\epsilon {\hat{E}}_m$	$\epsilon {\hat{E}}$	$\epsilon {\hat{F}}_m$	$\epsilon {\hat{F}}$
2	$\epsilon ^{\frac{1}{2}}{\check{T}}$	$\epsilon ^{\frac{1}{2}}{\check{R}}_m$	$\epsilon {\hat{R}}$	$\epsilon {\hat{A}}_m$	$\epsilon {\hat{A}}$	$\epsilon ^{\frac{1}{2}}{\check{C}}_m$	$\epsilon {\hat{C}}$	$\epsilon {\hat{B}}_m$	$\epsilon {\hat{B}}$	$\epsilon ^{\frac{1}{2}}{\check{S}}_m$	$\epsilon {\hat{S}}$	$\epsilon ^{\frac{1}{2}}{\check{E}}_m$	$\epsilon {\hat{E}}$	$\epsilon ^{\frac{1}{2}}{\check{F}}_m$	$\epsilon {\hat{F}}$
3	$\epsilon ^{\frac{1}{4}}{\bar{T}}$	$\epsilon ^{\frac{1}{4}}{\bar{R}}_m$	$\epsilon ^{\frac{1}{2}}{\bar{R}}$	$\epsilon {\hat{A}}_m$	$\epsilon {\hat{A}}$	$\epsilon ^{\frac{1}{4}}{\bar{C}}_m$	$\epsilon ^{\frac{1}{2}}{\bar{C}}$	$\epsilon {\hat{B}}_m$	$\epsilon {\hat{B}}$	$\epsilon ^{\frac{1}{4}}{\bar{S}}_m$	$\epsilon {\hat{S}}$	$\epsilon ^{\frac{1}{4}}{\bar{E}}_m$	$\epsilon {\hat{E}}$	$\epsilon ^{\frac{1}{4}}{\bar{F}}_m$	$\epsilon ^{\frac{1}{2}}{\bar{F}}$
4	$\epsilon ^{\frac{1}{6}}{\tilde{T}}$	$\epsilon ^{\frac{1}{6}}{\tilde{R}}_m$	$\epsilon ^{\frac{1}{3}}{\tilde{R}}$	$\epsilon {\hat{A}}_m$	$\epsilon {\hat{A}}$	$\epsilon ^{\frac{1}{6}}{\tilde{C}}_m$	$\epsilon ^{\frac{1}{3}}{\tilde{C}}$	$\epsilon {\hat{B}}_m$	$\epsilon {\hat{B}}$	$\epsilon ^{\frac{1}{6}}{\tilde{S}}_m$	$\epsilon ^{\frac{5}{6}}{\tilde{S}}$	$\epsilon ^{\frac{1}{6}}{\tilde{E}}_m$	$\epsilon ^{\frac{5}{6}}{\tilde{E}}$	$\epsilon ^{\frac{1}{6}}{\tilde{F}}_m$	$\epsilon ^{\frac{1}{3}}{\tilde{F}}$
5	$\epsilon ^{\frac{1}{8}}T'$	$\epsilon ^{\frac{1}{8}}R'_m$	$\epsilon ^{\frac{1}{4}}R'$	$\epsilon {\hat{A}}_m$	$\epsilon {\hat{A}}$	$\epsilon ^{\frac{1}{8}}C'_m$	$\epsilon ^{\frac{1}{4}}C'$	$\epsilon ^{\frac{7}{8}}B'_m$	$\epsilon {\hat{B}}$	$\epsilon ^{\frac{1}{8}}S'_m$	$\epsilon ^{\frac{3}{4}}S'$	$\epsilon ^{\frac{1}{8}}E'_m$	$\epsilon ^{\frac{3}{4}}E'$	$\epsilon ^{\frac{1}{8}}F'_m$	$\epsilon ^{\frac{1}{4}}F'$
6	$T^\dagger$	$R^\dagger _m$	$R^\dagger$	$\epsilon {\hat{A}}_m$	$\epsilon {\hat{A}}$	$C^\dagger _m$	$C^\dagger$	$\epsilon ^{\frac{1}{2}}B^\dagger _m$	$\epsilon ^{\frac{1}{2}}B^\dagger$	$S^\dagger _m$	$\epsilon ^{\frac{1}{2}}S^\dagger$	$E^\dagger _m$	$\epsilon ^{\frac{1}{2}}E^\dagger$	$F^\dagger _m$	$F^\dagger$
7	$\epsilon ^{-\frac{1}{2}}T^\ddagger$	$R^\dagger _m$	$\epsilon ^{-\frac{1}{2}}R^\ddagger$	$\epsilon {\hat{A}}_m$	$\epsilon ^{\frac{1}{2}}A^\ddagger$	$C^\dagger _m$	$\epsilon ^{-\frac{1}{2}}C^\ddagger$	$\epsilon ^{\frac{1}{2}}B^\dagger _m$	$B^\ddagger$	$S^\dagger _m$	$S^\ddagger$	$E^\dagger _m$	$E^\ddagger$	$F^\dagger _m$	$\epsilon ^{-\frac{1}{2}}F^\ddagger$
8	$\epsilon ^{-1}\phi ^{-1}\delta ^{-1} T^\diamond$	$R^\dagger _m$	$\epsilon ^{-1}\phi ^{-1}\delta ^{-1} R^\diamond$	$\epsilon {\hat{A}}_m$	$\epsilon ^{\frac{1}{2}}A^\ddagger$	$C^\dagger _m$	$\epsilon ^{-1}\phi ^{-1}\delta ^{-1} C^\diamond$	$\epsilon \phi \delta B^\diamond _m$	$\phi B^\diamond$	$S^\dagger _m$	$\epsilon ^{-\frac{1}{2}}\phi ^{-1}\delta ^{-1} S^\diamond$	$E^\dagger _m$	$\epsilon ^{-\frac{1}{2}}\phi ^{-1}\delta ^{-1} E^\diamond$	$\epsilon ^{-\frac{1}{2}}\phi \delta F^\diamond _m$	$\epsilon ^{-\frac{1}{2}}\delta F^\diamond$
9	$\epsilon ^{-1}T^+$	$R^\dagger _m$	$\epsilon ^{-1}R^+$	$\epsilon {\hat{A}}_m$	$\epsilon ^{\frac{1}{2}}A^\ddagger$	$C^\dagger _m$	$\epsilon ^{-1}C^+$	$\epsilon B^+_m$	$\phi B^\diamond$	$S^\dagger _m$	$\epsilon ^{-\frac{1}{2}} S^+$	$E^\dagger _m$	$\epsilon ^{-\frac{1}{2}} E^+$	$\epsilon ^{-\frac{1}{2}} F^+_m$	$\epsilon ^{-\frac{1}{2}}\delta F^\diamond$

## Time dependent asymptotic analysis

We now exploit asymptotic analyses to break down the full solution into smaller timescales to investigate how the system evolves over time. For an insight into various asymptotic techniques and methods, some used in this section, see (Kevorkian and Cole 2013). Variable scalings on each timescale are obtained by first finding the long-term behaviour of each variable on the previous timescale. Once this long-term or near blow up behaviour is found, we can identify the scalings based on how each variable behaves compared to our time variable *T*. For example if a nondimensionalised variable *G* behaves on the previous timescale as follows

$$\begin{aligned} G \sim T \text { as } T \rightarrow \infty , \end{aligned}$$

then to move to the next timescale we must scale *G* in the same way that we do for *T*. Throughout the next sections, we will draw comparisons between the numerical solutions and asymptotic approximations. In all figures asymptotic approximations will be shown in circles, whereas the numerical simulations will be shown as solid lines. We take $\epsilon =0.01$ unless otherwise stated. We also do not include all timescales in the following analysis, choosing only those that exhibit significant changes in behaviour. For a full breakdown of timescales, see (Youlden 2018).

### Asymptotic analysis of the wild-type dynamics

We begin with the wild-type case where RamR protein is not mutated (i.e $\mu =1$). We denote the variable scalings for each timescale in Table 4. Here the scalings are given in relation to the original nondimensionalised variables in (15). Throughout the simulations in this section, we take $\epsilon =0.01$, and all other parameters as unity unless otherwise stated. We do not plot every variable on each timescale, instead choosing to plot the variables involved with new terms entering the leading order balance.

#### Timescale 1: mRNA transcription

On this initial timescale all scalings must be scaled to $O(\epsilon )$ to reflect their initial conditions. Our system of equations rescaled for the first timescale is

$$\begin{aligned} {\frac{{ d{\hat{R}}}_{{m}}}{{ d{\hat{T}}}}}&=1-\epsilon {\hat{R}}_{{m}}, \\ {\frac{{ d{\hat{A}}}_{{m}}}{{ d{\hat{T}}}}}&={\frac{\epsilon {\hat{A}}}{ ( \epsilon {\hat{A}}+1 ) ( \epsilon {\hat{R}}+1 ) }}+\epsilon \alpha -\epsilon {\hat{A}}_{{m}}, \\ {\frac{{ d{\hat{C}}}_{{m}}}{{ d{\hat{T}}}}}&=\frac{\lambda }{\epsilon {\hat{A}}+\lambda }-\epsilon {\hat{C}}_{{m}}, \\ {\frac{{ d{\hat{B}}}_{{m}}}{{ d{\hat{T}}}}}&={\frac{\epsilon {\hat{A}}+\epsilon {\hat{S}}}{ ( 1+\epsilon {\hat{S}}+\epsilon {\hat{A}} ) ( 1+\epsilon {\hat{E}}+\epsilon {\hat{C}} ) }}-\epsilon {\hat{B}}_{{m}}, \\ {\frac{{ d{\hat{S}}}_{{m}}}{{ d{\hat{T}}}}}&=1-\epsilon {\hat{S}}_{{m}}, \\ {\frac{{ d{\hat{E}}}_{{m}}}{{ d{\hat{T}}}}}&=1-\epsilon {\hat{E}}_{{m}}, \\ {\frac{{ d{\hat{F}}}_{{m}}}{{ d{\hat{T}}}}}&={\frac{{ \eta }}{{ \eta }+\epsilon {\hat{E}}}}-\epsilon {\hat{F}}_{{m}},\\ {\frac{{ d{\hat{R}}}}{{ d{\hat{T}}}}}&=\mu \rho \,\epsilon {\hat{R}}_{{m}}-\epsilon ^2 \mu \varDelta {\hat{R}}, \\ {\frac{{ d{\hat{A}}}}{{ d{\hat{T}}}}}&=\theta \,\epsilon {\hat{A}}_{{m}}-\epsilon ^\frac{3}{2}\upsilon {\hat{A}} -\epsilon ^2\varDelta {\hat{A}}, \\ {\frac{{ d{\hat{C}}}}{{ d{\hat{T}}}}}&=\gamma \,\epsilon {\hat{C}}_{{m}}-\epsilon ^2\varDelta {\hat{C}}, \\ {\frac{{ d{\hat{B}}}}{{ d{\hat{T}}}}}&=\beta \,\epsilon {\hat{B}}_{{m}}-\epsilon ^2\varDelta {\hat{B}}, \\ {\frac{{ d{\hat{S}}}}{{ d{\hat{T}}}}}&=\epsilon ^{3/2}\sigma \, {\hat{S}}_{{m}}-\epsilon ^2\varDelta {\hat{S}}, \\ {\frac{{ d{\hat{E}}}}{{ d{\hat{T}}}}}&=\epsilon ^{3/2}\xi \, {\hat{E}}_{{m}}-\epsilon ^2\varDelta {\hat{E}}, \\ {\frac{{ d{\hat{F}}}}{{ d{\hat{T}}}}}&={\frac{\epsilon {\hat{F}}_{{m}}}{\epsilon \omega {\hat{B}}+1}}-\epsilon ^2\varDelta {\hat{F}}. \end{aligned}$$

By finding the leading order balance of this system, we can reduce the system while maintaining the dominant behaviour on this timescale. Solving the reduced model subject to the initial conditions gives the following asymptotic approximations on this timescale

37 $$\begin{aligned} {\hat{R}}_m&={\hat{T}}+R_{m0},&{\hat{R}}&=R_0,&{\hat{A}}_m&=A_{m0},&{\hat{A}}&=A_0, \nonumber \\ {\hat{C}}_m&={\hat{T}}+C_{m0},&{\hat{C}}&=C_0,&{\hat{B}}_m&=B_{m0},&{\hat{B}}&=B_0, \nonumber \\ {\hat{S}}_m&={\hat{T}}+S_{m0},&{\hat{S}}&=S_0,&{\hat{E}}_m&={\hat{T}}+E_{m0},&{\hat{E}}&=E_0, \nonumber \\ {\hat{F}}_m&={\hat{T}}+F_{m0},&{\hat{F}}&=F_0. \end{aligned}$$

**Fig. 5:**
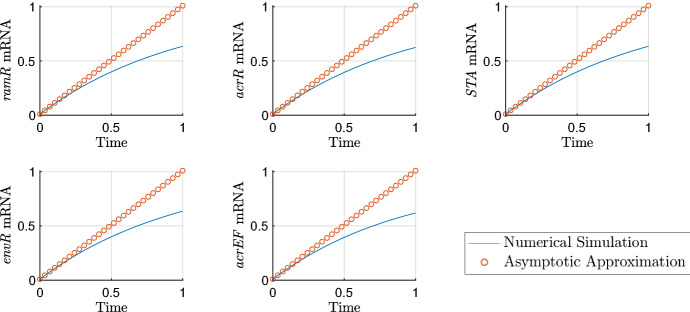
Asymptotic approximations on timescale 1 ($\epsilon =0.01$). On this timescale, time is $O(\epsilon )$, so we expect the asymptotics to be accurate around $T=\epsilon$

We plot the asymptotic approximations of those variables that evolve on this timescale against the numerical solutions in Fig. 5. As expected, we see the transcription of various gene’s mRNA occurring first with protein levels remaining at their initial value. The transcription of *ramA* and *acrAB* mRNA are currently not active due to there being insufficient levels of activator protein bound to their promoter sites to achieve any level of transcription at leading order.

#### Timescale 3: protein translation

We omit details of timescale 2 (where protein translation begins for a small number of transcribed mRNAs) for brevity. On this third timescale protein translation occurs at leading order for all proteins for which transcription of their corresponding mRNA occurred on timescale 1. The system of equations rescaled for the third timescale is

$$\begin{aligned} {\frac{{ d{\bar{R}}}_{{m}}}{{ d{\bar{T}}}}}&=1-\epsilon ^\frac{1}{4} {\bar{R}}_{{m}}, \\ {\frac{{ d{\hat{A}}}_{{m}}}{{ d{\bar{T}}}}}&={\frac{\epsilon ^\frac{1}{4} {\hat{A}}}{ ( \epsilon {\hat{A}}+1 ) ( \epsilon ^\frac{1}{2} {\bar{R}}+1 ) }}+\epsilon ^\frac{1}{4}\alpha -\epsilon ^\frac{1}{4}{\hat{A}}_{{m}}, \\ {\frac{{ d{\bar{C}}}_{{m}}}{{ d{\bar{T}}}}}&=\frac{\lambda }{\epsilon {\hat{A}}+\lambda }-\epsilon ^\frac{1}{4}{\bar{C}}_{{m}}, \\ {\frac{{ d{\hat{B}}}_{{m}}}{{ d{\bar{T}}}}}&={\frac{\epsilon ^\frac{1}{4} {\hat{A}}+\epsilon ^\frac{1}{4} {\hat{S}}}{ ( 1+\epsilon {\hat{S}}+\epsilon {\hat{A}} ) ( 1+\epsilon {\hat{E}}+\epsilon ^\frac{1}{2} {\bar{C}} ) }}-\epsilon ^\frac{1}{4}{\hat{B}}_{{m}}, \\ {\frac{{ d{\bar{S}}}_{{m}}}{{ d{\bar{T}}}}}&=1-\epsilon ^\frac{1}{4} {\bar{S}}_{{m}}, \\ {\frac{{ d{\bar{E}}}_{{m}}}{{ d{\bar{T}}}}}&=1-\epsilon ^\frac{1}{4} {\bar{E}}_{{m}}, \\ {\frac{{ d{\bar{F}}}_{{m}}}{{ d{\bar{T}}}}}&={\frac{{ \eta }}{{ \eta }+\epsilon {\hat{E}}}}-\epsilon ^\frac{1}{4} {\bar{F}}_{{m}},\\ {\frac{{ d{\bar{R}}}}{{ d{\bar{T}}}}}&=\mu \rho \, {\bar{R}}_{{m}}-\epsilon ^\frac{5}{4} \mu \varDelta {\bar{R}}, \\ {\frac{{ d{\hat{A}}}}{{ d{\bar{T}}}}}&=\epsilon ^\frac{1}{4}\theta \, {\hat{A}}_{{m}}-\epsilon ^{\frac{3}{4}}\upsilon \, {\hat{A}}-\epsilon ^\frac{5}{4}\varDelta {\hat{A}}, \\ {\frac{{ d{\bar{C}}}}{{ d{\bar{T}}}}}&= \gamma \, {\bar{C}}_{{m}}-\epsilon ^\frac{5}{4}\varDelta {\bar{C}}, \\ {\frac{{ d{\hat{B}}}}{{ d{\bar{T}}}}}&=\epsilon ^\frac{1}{4} \beta \, {\hat{B}}_{{m}}-\epsilon ^\frac{5}{4}\varDelta {\hat{B}}, \\ {\frac{{ d{\hat{S}}}}{{ d{\bar{T}}}}}&=\sigma \, {\bar{S}}_{{m}}-\epsilon ^\frac{5}{4}\varDelta {\hat{S}}, \\ {\frac{{ d{\hat{E}}}}{{ d{\bar{T}}}}}&=\xi \, {\bar{E}}_{{m}}-\epsilon ^\frac{5}{4}\varDelta {\hat{E}}, \\ {\frac{{ d{\bar{F}}}}{{ d{\bar{T}}}}}&={\frac{{\bar{F}}_{{m}}}{\epsilon \omega {\hat{B}}+1}}-\epsilon ^\frac{5}{4}\varDelta {\bar{F}}. \end{aligned}$$

Taking the leading order balance, solving and matching to the long-term dominant behaviour on the previous timescale gives the following asymptotic approximations

38 $$\begin{aligned} {\bar{R}}_m&={\bar{T}},&{\bar{R}}&=\frac{\mu \rho }{2}{\bar{T}}^2,&{\hat{A}}_m&=A_{m0},&{\hat{A}}&=A_0, \nonumber \\ {\bar{C}}_m&={\bar{T}},&{\bar{C}}&=\frac{\gamma }{2} {\bar{T}}^2,&{\hat{B}}_m&=B_{m0},&{\hat{B}}&=B_0, \nonumber \\ {\bar{S}}_m&={\bar{T}},&{\hat{S}}&=\frac{\sigma }{2} {\bar{T}}^2+S_0,&{\bar{E}}_m&={\bar{T}},&{\hat{E}}&=\frac{\xi }{2} {\bar{T}}^2+E_0, \nonumber \\ {\bar{F}}_m&={\bar{T}},&{\bar{F}}&=\frac{1}{2}{\bar{T}}^2. \end{aligned}$$

**Fig. 6:**
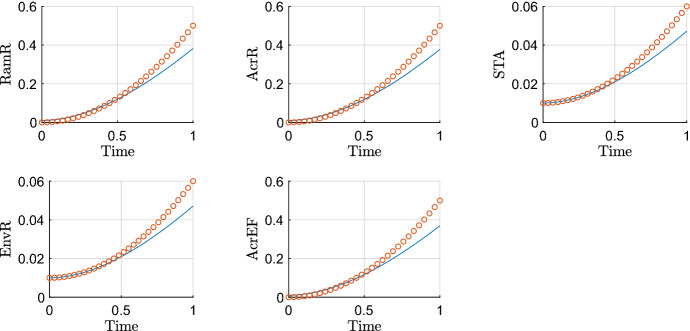
Asymptotic approximations on timescale 3 ($\epsilon =0.01$). On this timescale, time is $O(\epsilon ^\frac{1}{4})$, so we expect the asymptotics to be accurate around $T=\epsilon ^\frac{1}{4}\approx 0.3162$

We plot asymptotic approximations of protein variables that evolve on this timescale against the numerical solutions in Fig. 6. On this timescale, we see the translation of all mRNAs that were previously transcribed on the earlier timescales. This makes logical sense as we expect rapid translation in response to changes at the transcriptional level.

#### Timescale 5: AcrAB translation

We omit timescale 4 (where only *acrAB* mRNA transcription takes place) for brevity. On this fifth timescale the translation term for AcrAB appears in the leading order balance, thus our system of equations rescaled for the fifth timescale is

$$\begin{aligned} {\frac{{ dR_m'}}{{ dT'}}}&=1-\epsilon ^\frac{1}{8}R_m', \\ {\frac{{ d{\hat{A}}}_{{m}}}{{ dT'}}}&={\frac{\epsilon ^\frac{1}{8} {\hat{A}}}{ ( \epsilon {\hat{A}}+1 ) ( \epsilon ^\frac{1}{4} R'+1 ) }}+\epsilon ^\frac{1}{8}\alpha -\epsilon ^\frac{1}{8} {\hat{A}}_{{m}}, \\ {\frac{{ dC_m'}}{{ dT'}}}&=\frac{\lambda }{\epsilon {\hat{A}}+\lambda }-\epsilon ^\frac{1}{8}C_m', \\ {\frac{{ dB}_{{m}}'}{{ dT'}}}&={\frac{\epsilon ^\frac{1}{4} {\hat{A}}+S'}{ ( 1+\epsilon ^\frac{3}{4} S'+\epsilon {\hat{A}} ) ( 1+\epsilon ^\frac{3}{4} E'+\epsilon ^\frac{1}{4} C' ) }}-\epsilon ^\frac{1}{8} B_m', \\ {\frac{{ dS_m'}}{{ dT'}}}&=1-\epsilon ^\frac{1}{8} S_m', \\ {\frac{{ dE_m'}}{{ dT'}}}&=1-\epsilon ^\frac{1}{8} E_m', \\ {\frac{{ dF_m'}}{{ dT'}}}&={\frac{{ \eta }}{{ \eta }+\epsilon ^\frac{3}{4} E'}}-\epsilon ^\frac{1}{8} F_m',\\ {\frac{{ dR'}}{{ dT'}}}&= \mu \rho \,R_m'-\epsilon ^\frac{9}{8} \mu \varDelta \,R', \\ {\frac{{ d{\hat{A}}}}{{ dT'}}}&=\epsilon ^\frac{1}{8} \theta \, {\hat{A}}_{{m}}-\epsilon ^\frac{5}{8}\upsilon \, {\hat{A}}-\epsilon ^\frac{9}{8}\varDelta {\hat{A}}, \\ {\frac{{ dC'}}{{ dT'}}}&= \gamma \, C_m'-\epsilon ^\frac{9}{8}\varDelta C', \\ \frac{d\hat{B}}{ dT'}&=\beta \, B_m'-\epsilon ^\frac{9}{8}\varDelta \,\hat{B},\\ {\frac{{ dS'}}{{ dT'}}}&=\sigma \, S_m'-\epsilon ^\frac{9}{8}\varDelta \,S', \\ {\frac{{ dE'}}{{ dT'}}}&=\xi \, E_m'-\epsilon ^\frac{9}{8}\varDelta \,E', \\ {\frac{{ dF'}}{{ dT'}}}&={\frac{ F_m'}{\epsilon \omega {\hat{B}}+1}}-\epsilon ^\frac{9}{8}\varDelta F'. \end{aligned}$$

We solve the leading order balance and matching to the long-term dominant behaviour on the previous timescale gives the following asymptotic approximations

39 $$\begin{aligned} R_m'&=T',&R'&=\frac{\mu \rho }{2}T'^2,&{\hat{A}}_m&=A_{m0},&{\hat{A}}&=A_0, \nonumber \\ C_m'&=T',&C'&=\frac{\gamma }{2} T'^2,&B_m'&=\frac{\sigma }{6} T'^3,&{\hat{B}}&=\frac{\beta \sigma }{24} T'^4+B_0, \nonumber \\ S_m'&=T',&S'&=\frac{\sigma }{2} T'^2,&E_m'&=T',&E'&=\frac{\xi }{2} T'^2, \nonumber \\ F_m'&=T',&F'&=\frac{1}{2}T'^2. \end{aligned}$$

**Fig. 7:**
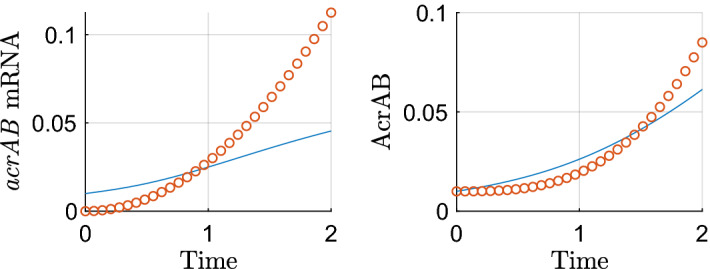
Asymptotic approximations on timescale 5 ($\epsilon =0.01$). On this timescale, time is $O(\epsilon ^\frac{1}{8})$, so we expect the asymptotics to be accurate around $T=\epsilon ^\frac{1}{8}\approx 0.5623$. We could eliminate the disparity by matching to more than the blow up behaviour on the previous timescale, however for simplicity in solutions for the latter timescales, we have opted not to do so

The asymptotic approximations for *acrAB* are plotted against the full solution in Fig. 7. In this timescale, we have translation of AcrAB at the leading order, we note that this is being driven here by the secondary TA. Here we exhibit both mRNA and protein to exhibit the new dominant behaviour emerging in both timescales 4 and 5. We note there is disparity between the approximations and numerics. We could eliminate this by matching to additional orders of behaviour on the previous timescale, however for simplicity in solutions for the latter timescales, we have opted not to do so.

#### Timescale 6: mRNA degradation and full protein translation

For this timescale, mRNA degradation and expression of *ramA* enters the leading order balance, our system of equations rescaled for the sixth timescale is

$$\begin{aligned} {\frac{{ dR_m^\dagger }}{{ dT^\dagger }}}&=1-R_m^\dagger , \\ {\frac{{ d{\hat{A}}}_{{m}}}{{ dT^\dagger }}}&={\frac{{\hat{A}}}{ (\epsilon {\hat{A}}+1) (R^\dagger +1) }}+\alpha - {\hat{A}}_{{m}}, \\ {\frac{{ dC_m^\dagger }}{{ dT^\dagger }}}&=\frac{\lambda }{\epsilon {\hat{A}}+\lambda }-C_m^\dagger , \\ {\frac{{ dB_m^\dagger }}{{ dT^\dagger }}}&={\frac{\epsilon ^\frac{1}{2} {\hat{A}}+S^\dagger }{ ( 1+\epsilon ^\frac{1}{2} S^\dagger +\epsilon {\hat{A}} ) ( 1+\epsilon ^\frac{1}{2} E^\dagger + C^\dagger ) }}- B_m^\dagger , \\ {\frac{{ dS_m^\dagger }}{{ dT^\dagger }}}&=1-S_m^\dagger , \\ {\frac{{ dE_m^\dagger }}{{ dT^\dagger }}}&=1-E_m^\dagger , \\ {\frac{{ dF_m^\dagger }}{{ dT^\dagger }}}&={\frac{{ \eta }}{{ \eta }+\epsilon ^\frac{1}{2} E^\dagger }}- F_m^\dagger ,\\ {\frac{{ dR^\dagger }}{{ dT^\dagger }}}&= \mu \rho \, R_m^\dagger -\epsilon \mu \varDelta \,R^\dagger , \\ {\frac{{ d{\hat{A}}}}{{ dT^\dagger }}}&=\theta \, {\hat{A}}_{{m}}-\epsilon ^{\frac{1}{2}}\upsilon \, {\hat{A}}-\epsilon \varDelta {\hat{A}}, \\ {\frac{{ dC^\dagger }}{{ dT^\dagger }}}&= \gamma \, C_m^\dagger -\epsilon \varDelta C^\dagger , \\ {\frac{{ dB^\dagger }}{{ dT^\dagger }}}&= \beta \, B_m^\dagger -\epsilon \varDelta \,B^\dagger , \\ {\frac{{ dS^\dagger }}{{ dT^\dagger }}}&=\sigma \, S_m^\dagger -\epsilon \varDelta \,S^\dagger , \\ {\frac{{ dE^\dagger }}{{ dT^\dagger }}}&=\xi \, E_m^\dagger -\epsilon \varDelta \,E^\dagger , \\ {\frac{{ dF^\dagger }}{{ dT^\dagger }}}&={\frac{ F_m^\dagger }{\epsilon ^\frac{1}{2} \omega B^\dagger +1}}-\epsilon \varDelta F^\dagger . \end{aligned}$$

Solving the leading order balance and matching to the long-term dominant behaviour on the previous timescale gives the following asymptotic approximations

40 $$\begin{aligned} R_m^\dagger&=1-e^{-T^\dagger },&R^\dagger&=\mu \rho \left( T^\dagger -e^{-T^\dagger } -1\right) ,&C_m^\dagger&=1-e^{-T^\dagger }, \nonumber \\ B_m^\dagger&=\frac{\sigma T^\dagger }{\gamma T^\dagger +1},&C^\dagger&=\gamma \left( T^\dagger -e^{-T^\dagger } -1\right) ,&B^\dagger&={\frac{\beta \sigma }{\gamma }}\left( 1-{\frac{\ln \left( T^\dagger \gamma + 1 \right) }{{\gamma }}}\right) , \nonumber \\ S_m^\dagger&=1-e^{-T^\dagger },&S^\dagger&=\sigma \left( T^\dagger -e^{-T^\dagger } -1\right) ,&E_m^\dagger&=1-e^{-T^\dagger }, \nonumber \\ F_m^\dagger&=1-e^{-T^\dagger },&E^\dagger&=\xi \left( T^\dagger -e^{-T^\dagger } -1\right) ,&F^\dagger&=T^\dagger -e^{-T^\dagger } -1, \end{aligned}$$

while the behaviour for both *ramA* mRNA (${\hat{A}}_m$) and RamA protein (${\hat{A}}$) depends on the relationship between the parameters $\mu$,$\theta$ and $\rho$. If $\dfrac{\theta }{\mu \rho }\ne 1$ we have

41 $$\begin{aligned} {\hat{A}}_m&=\frac{ \left( \mu \rho A_{{0}} - \theta \left( \alpha +A_{{0}} \right) \right) \left( T^\dagger \mu \rho +1 \right) ^{{\frac{\theta }{\mu \rho }}}+ \left( T^\dagger \mu \rho +1 \right) \theta \,\alpha }{(\mu \rho -\theta )(\mu \rho T^\dagger +1)},&\end{aligned}$$

42 $$\begin{aligned} {\hat{A}}&=\frac{ \left( \mu \rho A_{{0}} - \theta \left( \alpha +A_{{0}} \right) \right) \left( T^\dagger \mu \rho +1 \right) ^{{\frac{\theta }{\mu \rho }}}+ \left( T^\dagger \mu \rho +1 \right) \theta \,\alpha }{\mu \rho -\theta }. \end{aligned}$$

In this case we have different long term blow up behaviour depending on whether $\dfrac{\theta }{\mu \rho }<1$ or $\dfrac{\theta }{\mu \rho }>1$, detailed below. For the case where $\dfrac{\theta }{\mu \rho }= 1$ we have

43 $$\begin{aligned} {\hat{A}}_m&=\alpha \,\ln \left( T^\dagger +1 \right) +A_{{0}}+\alpha ,&{\hat{A}}&= \left( \alpha \,\ln \left( T^\dagger +1 \right) +A_{{0}} \right) \left( T^\dagger +1 \right) , \end{aligned}$$

here we have taken the case where $\theta =1, \mu =1, \rho =1$ for simplicity of displaying the solutions.

We can see that we have three cases of long-term behaviour for both *ramA* mRNA (${\hat{A}}_m$) and RamA protein (${\hat{A}}$), we exhibit the relation between this long-term behaviour and our parameter groupings as follows

44 $$\begin{aligned}&{\hat{A}}_m \sim {\left\{ \begin{array}{ll} C_A &{} \text {for } \dfrac{\theta }{\mu \rho }<1,\\ \ln (T^\dagger ) &{} \text {for } \dfrac{\theta }{\mu \rho }=1,\\ {T^\dagger }^{\frac{\theta }{\mu \rho }-1} &{} \text {for } \dfrac{\theta }{\mu \rho }>1, \end{array}\right. } \end{aligned}$$

45 $$\begin{aligned}&{\hat{A}} \sim {\left\{ \begin{array}{ll} T^\dagger ,&{} \text {for } \dfrac{\theta }{\mu \rho }<1,\\ T^\dagger \ln (T^\dagger ) &{} \text {for } \dfrac{\theta }{\mu \rho }=1,\\ {T^\dagger }^{\frac{\theta }{\mu \rho }} &{} \text {for } \dfrac{\theta }{\mu \rho }>1, \end{array}\right. } \end{aligned}$$

where here $C_A$ is a constant. We note that the parameter $\theta$ relates to RamA production, whilst $\rho$ relates to RamR production. Since RamR is a repressor of *ramA* expression, we might expect its rate of production to dominate, thus we note that the case $\dfrac{\theta }{\mu \rho }<1$ is the most biologically plausible and use the resulting behaviour to move to the next timescale. For all future numerical simulations, we set $\theta =0.5$ to satisfy this inequality.

**Fig. 8:**
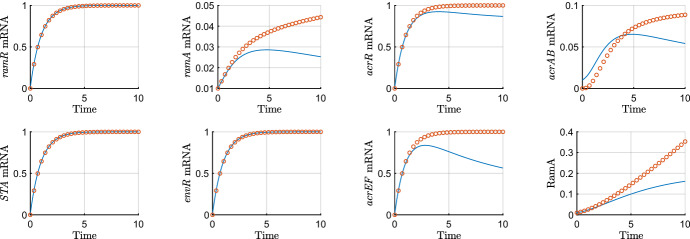
Asymptotic approximations on timescale 6 ($\epsilon =0.01$). On this timescale, time is *O*(1), so we expect the asymptotics to be accurate around $T=1$. Note we depict the simulations over a longer period of time than earlier timescales

We plot these asymptotic approximations against the full solution in Fig. 8. This is the first timescale where the *ramA* gene is expressed at leading order, this is due to there being little RamA protein in the system to activate its own expression. We note we have transcription of *ramA* mRNA and translation of RamR coming into this timescale. In addition to this we have degradation terms for all mRNAs, this is causing the mRNAs to level off and reach steady state. In addition to this, the local repressor of *acrAB* (AcrR) is bound to the operator site of *acrAB* which is in effect limiting the transcription of this gene. For *acrAB* mRNA we have a slight mismatch of the approximation to the solution, we could prevent this by matching to lower orders of behaviour on the previous timescale. However, we have chosen not to do this for simplicity of solutions on this and further timescales.

#### Timescale 7: degradation of RamA, inhibition of *acrAB* and *acrEF*

For this timescale, we have a change of the terms involved in transcription of *acrAB* and *acrEF*, with new terms emerging at leading order. We also have RamA degradation entering the leading order balance. Our system of equations rescaled for the seventh timescale is

$$\begin{aligned} \epsilon ^\frac{1}{2}{\frac{dR_m^\dagger }{{ dT^\ddagger }}}&=1-R_m^\dagger , \\ \epsilon {\frac{{ d{\hat{A}}}_{{m}}}{{ dT^\ddagger }}}&={\frac{A^\ddagger }{ (\epsilon ^\frac{1}{2} A^\ddagger +1) (\epsilon ^{-\frac{1}{2}}R^\ddagger +1) }}+\epsilon ^\frac{1}{2}\alpha -\epsilon ^\frac{1}{2} {\hat{A}}_{{m}}, \\ \epsilon ^\frac{1}{2}{\frac{dC_m^\dagger }{{ dT^\ddagger }}}&=\frac{\lambda }{\epsilon ^\frac{1}{2} A^\ddagger +\lambda }-C_m^\dagger , \\ \epsilon {\frac{dB_m^\dagger }{{ dT^\ddagger }}}&={\frac{\epsilon ^\frac{1}{2} A^\ddagger +S^\ddagger }{ ( 1+S^\ddagger +\epsilon ^\frac{1}{2}A^\ddagger ) ( 1+E^\ddagger + \epsilon ^{-\frac{1}{2}}C^\ddagger ) }}-\epsilon ^\frac{1}{2} B_m^\dagger , \\ \epsilon ^\frac{1}{2}{\frac{dS_m^\dagger }{{ dT^\ddagger }}}&=1-S_m^\dagger , \\ \epsilon ^\frac{1}{2}{\frac{dE_m^\dagger }{{ dT^\ddagger }}}&=1-E_m^\dagger , \\ \epsilon ^\frac{1}{2}{\frac{dF_m^\dagger }{{ dT^\ddagger }}}&={\frac{{ \eta }}{{ \eta }+E^\ddagger }}- F_m^\dagger ,\\ {\frac{{ dR^\ddagger }}{{ dT^\ddagger }}}&= \mu \rho \, R_m^\dagger -\epsilon ^\frac{1}{2} \mu \varDelta \,R^\ddagger , \\ {\frac{{ dA^\ddagger }}{{ dT^\ddagger }}}&= \theta \, {\hat{A}}_{{m}}-\upsilon \, A^\ddagger -\epsilon ^\frac{1}{2}\varDelta A^\ddagger , \\ {\frac{{ dC^\ddagger }}{{ dT^\ddagger }}}&= \gamma \, C_m^\dagger -\epsilon ^\frac{1}{2}\varDelta C^\ddagger , \\ {\frac{{ dB^\ddagger }}{{ dT^\ddagger }}}&= \beta \, B^\ddagger _{{m}}-\epsilon ^\frac{1}{2}\varDelta \,B^\ddagger , \\ {\frac{{ dS^\ddagger }}{{ dT^\ddagger }}}&=\sigma \, S_m^\dagger -\epsilon ^\frac{1}{2}\varDelta \,S^\ddagger , \\ {\frac{{ dE^\ddagger }}{{ dT^\ddagger }}}&=\xi \, E_m^\dagger -\epsilon ^\frac{1}{2}\varDelta \,E^\ddagger , \\ {\frac{{ dF^\ddagger }}{{ dT^\ddagger }}}&={\frac{ F_m^\dagger }{\omega B^\ddagger +1}}-\epsilon ^\frac{1}{2}\varDelta F^\ddagger . \end{aligned}$$

Matching the solutions of the leading order balance to the long-term dominant behaviour on the previous timescale gives the following asymptotic approximations

46 $$\begin{aligned}&\displaystyle R_m^\dagger =1,\qquad R^\ddagger =\mu \rho T^\ddagger ,\qquad {\hat{A}}_m=\frac{ \alpha \upsilon \mu \rho T^\ddagger }{\upsilon \mu \rho T^\ddagger -\theta },\qquad A^\ddagger =\frac{\theta \alpha \mu \rho T^\ddagger }{\upsilon \mu \rho T^\ddagger -\theta }, \nonumber \\&\displaystyle C_m^\dagger =1,\qquad C^\ddagger =\gamma T^\ddagger ,\qquad B_m^\dagger =\frac{\sigma }{\gamma (\sigma T^\ddagger +1)},\qquad S_m^\dagger =1,\nonumber \\&\displaystyle S^\ddagger =\sigma T^\ddagger ,\qquad E_m^\dagger =1,\qquad E^\ddagger =\xi T^\ddagger ,\qquad F_m^\dagger =\frac{\eta }{\eta +\xi T^\ddagger },\nonumber \\&\displaystyle B^\ddagger =\frac{\beta }{\gamma }\ln (\sigma T^\ddagger +1),\qquad F^\ddagger =\frac{\eta \gamma }{\beta \omega \xi }\ln (\beta \omega \ln (\xi T^\ddagger +\eta )+\gamma ). \end{aligned}$$

**Fig. 9:**
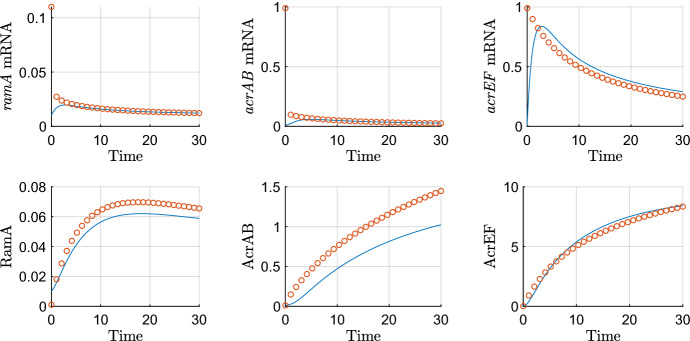
Asymptotic approximations on timescale 6 ($\epsilon =0.01$). On this timescale, time is $O(\epsilon ^{-\frac{1}{2}})$, so we expect the asymptotics to be accurate around $T=\epsilon ^{-\frac{1}{2}}=10$. Note we depict the simulations over a longer period of time than earlier timescales

We plot asymptotic approximations of those variables that evolve on this timescale against the numerical solutions in Fig. 9. On this timescale, we have repressor proteins dominating the transcription terms for *acrAB* and *acrEF*. With lower levels of transcription, degradation dominates and the concentrations of the mRNAs lower. We note that in this wild-type case RamA production occurs late compared to other proteins (starting on the previous timescale) and is quickly degraded to achieve only low levels in comparison to other proteins in the system. On this timescale, all other mRNAs have reached steady state and AcrAB and AcrEF grow logarithmically.

#### Timescale 9: final timescale, protein degradation

We omit the eighth timescale (which scales away from the logarithmic behaviour of the efflux pump genes) for brevity. On this timescale degradation for all proteins that were not already at steady state emerge in the leading order balance. Thus using these scalings, our system of equations rescaled for the ninth and final timescale is

$$\begin{aligned} \epsilon {\frac{dR^\dagger _{{m}}}{{ dT^+}}}&=1-R^\dagger _{{m}}, \\ \epsilon ^\frac{3}{2}{\frac{{ d{\hat{A}}}_{{m}}}{{ dT^+}}}&={\frac{A^\ddagger }{ ( \epsilon ^\frac{1}{2}A^\ddagger +1) (\epsilon ^{-1}R^++1) }}+\epsilon ^\frac{1}{2}\alpha -\epsilon ^\frac{1}{2}{\hat{A}}_{{m}}, \\ \epsilon {\frac{dC^\dagger _{{m}}}{{ dT^+}}}&=\frac{\lambda }{ A^++\lambda }-C^\dagger _{{m}}, \\ \epsilon ^2{\frac{dB^+_{{m}}}{{ dT^+}}}&={\frac{ A^++\epsilon ^{-\frac{1}{2}}S^+}{ ( 1+\epsilon ^{-\frac{1}{2}}S^++A^+ ) ( 1+\epsilon ^{-\frac{1}{2}}E^++ \epsilon ^{-1}C^+ ) }}-\epsilon B^+_{{m}}, \\ \epsilon {\frac{dS^\dagger _{{m}}}{{ dT^+}}}&=1-S^\dagger _{{m}}, \\ \epsilon {\frac{dE^\dagger _{{m}}}{{ dT^+}}}&=1-E^\dagger _{{m}}, \\ \epsilon ^\frac{3}{2}{\frac{dF^+_{{m}}}{{ dT^+}}}&={\frac{{ \eta }}{{ \eta }+\epsilon ^{-\frac{1}{2}}E^+}}- \epsilon ^{\frac{1}{2}} F^+_{{m}},\\ {\frac{{ dR^+}}{{ dT^+}}}&= \mu \rho \, R^\dagger _{{m}}- \mu \varDelta \,R^+, \\ \epsilon ^\frac{1}{2}{\frac{{ dA^\ddagger }}{{ dT^+}}}&=\theta \, {\hat{A}}_{{m}}-\upsilon \,A^\ddagger -\epsilon ^\frac{1}{2}\varDelta A^\ddagger , \\ {\frac{{ dC^+}}{{ dT^+}}}&= \gamma \, C^\dagger _{{m}}-\varDelta C^+, \\ {\frac{{ dB^\diamond }}{{ dT^+}}}&= \beta \phi \, B^+_{{m}}-\varDelta \,B^\diamond , \\ {\frac{{ dS^+}}{{ dT^+}}}&=\sigma \, S^\dagger _{{m}}-\varDelta \,S^+, \\ {\frac{{ dE^+}}{{ dT^+}}}&=\xi \, E^\dagger _{{m}}-\varDelta \,E^+, \\ {\frac{{ dF^\diamond }}{{ dT^+}}}&=\frac{\phi \delta F_m^+}{(\omega B^\diamond +\phi )}-\varDelta F^\diamond . \end{aligned}$$

Here, the terms $\phi =\ln (1/\epsilon )^{-1}$ and $\delta =\ln (\ln (1/\epsilon ))^{-1}$ have emerged from logarithmic behaviour on previous timescales. With the value of $\epsilon =0.01$, these terms are effectively *O*(1), so we include them in the leading order balance. Matching to the long-term dominant behaviour on the previous timescale gives the following asymptotic approximations

47 $$\begin{aligned}&\displaystyle R_m^\dagger =1,\qquad R^+=\frac{\mu \rho }{\varDelta }(1-e^{-\varDelta T^+}),\qquad {\hat{A}}_m=\alpha , \nonumber \\&\displaystyle A^\ddagger =\frac{\theta \alpha }{\upsilon },\qquad C_m^\dagger =1,\qquad C^+=\frac{\gamma }{\varDelta }(1-e^{-\varDelta T^+}), \nonumber \\&\displaystyle S_m^\dagger =1,\qquad S^+=\frac{\sigma }{\varDelta }(1-e^{-\varDelta T^+}),\qquad E_m^\dagger =1, \nonumber \\&\displaystyle E^+=\frac{\xi }{\varDelta }(1-e^{-\varDelta T^+}), B_m^+=\frac{\varDelta }{\gamma (1-e^{-\varDelta T^+})},\qquad F_m^+=\frac{\eta \varDelta }{\xi (1-e^{-\varDelta T^+})},\nonumber \\&\displaystyle B^\diamond = \frac{\phi \beta }{\gamma }(1+\ln (\gamma (e^{\varDelta T^+}-1))e^{-\varDelta T^+}),\qquad F^\diamond =\frac{\delta \eta \gamma }{\xi (\beta \omega +\gamma )}(1+\frac{\gamma }{\beta \omega }e^{-\varDelta T^+}).\nonumber \\ \end{aligned}$$

**Fig. 10:**
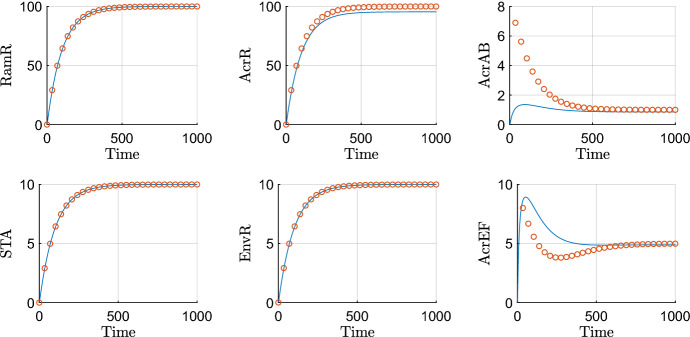
Asymptotic approximations on timescale 6 ($\epsilon =0.01$). On this timescale, time is $O(\epsilon ^{-1})$, so we expect the asymptotics to be accurate around and beyond $T=\epsilon ^{-1}$. Note we depict the simulations over a larger period of time than earlier timescales

We plot asymptotic approximations of those variables that evolve on this timescale against the numerical solutions in Fig. 10. On this final timescale, we see all proteins reaching a steady state as their degradation terms appear at leading order. We note that the approximated steady states match closely to the numerics, thus we should be able to draw strong conclusions by performing steady state analysis on the approximations, shown in Sect. 4.3. This concludes the wild-type asymptotic analysis. In summation, we have broken down the system into nine timescales. For each of these timescales we have obtained full analytical solutions.

### Asymptotic analysis of the mutant dynamics

In this section we take the case where RamR protein is mutated (i.e $\mu =0$). We denote the scalings we must take in order to reach each timescale in Table 5. Throughout the simulations in this section, we take $\epsilon =0.01$, and all other parameters as unity unless otherwise stated. We omit the first five timescales in the below for brevity as they follow closely from the same timescales in the wild-type asymptotics without translation of RamR protein.

#### Timescale 6: mRNA degradation and full protein translation

**Table 5:** The scalings for each variable required on each timescale for the mutant asymptotic analysis. Here we have $\kappa =\frac{1}{2}(-1+\sqrt{1+4\theta })$, $\phi =\ln (1/\epsilon )^{-1}$ and $\delta =\ln (\ln (1/\epsilon ))^{-1}$ as explained in the timescales in which they feature

Timescale	Variable														
	*T*	$R_m$	*R*	$A_m$	*A*	$C_m$	*C*	$B_m$	*B*	$S_m$	*S*	$E_m$	*E*	$F_m$	*F*
1	$\epsilon {\hat{T}}$	$\epsilon {\hat{R}}_m$	$\epsilon {\hat{R}}$	$\epsilon {\hat{A}}_m$	$\epsilon {\hat{A}}$	$\epsilon {\hat{C}}_m$	$\epsilon {\hat{C}}$	$\epsilon {\hat{B}}_m$	$\epsilon {\hat{B}}$	$\epsilon {\hat{S}}_m$	$\epsilon {\hat{S}}$	$\epsilon {\hat{E}}_m$	$\epsilon {\hat{E}}$	$\epsilon {\hat{F}}_m$	$\epsilon {\hat{F}}$
2	$\epsilon ^{\frac{1}{2}}{\check{T}}$	$\epsilon ^{\frac{1}{2}}{\check{R}}_m$	$\epsilon {\hat{R}}$	$\epsilon {\hat{A}}_m$	$\epsilon {\hat{A}}$	$\epsilon ^{\frac{1}{2}}{\check{C}}_m$	$\epsilon {\hat{C}}$	$\epsilon {\hat{B}}_m$	$\epsilon {\hat{B}}$	$\epsilon ^{\frac{1}{2}}{\check{S}}_m$	$\epsilon {\hat{S}}$	$\epsilon ^{\frac{1}{2}}{\check{E}}_m$	$\epsilon {\hat{E}}$	$\epsilon ^{\frac{1}{2}}{\check{F}}_m$	$\epsilon {\hat{F}}$
3	$\epsilon ^{\frac{1}{4}}{\bar{T}}$	$\epsilon ^{\frac{1}{4}}{\bar{R}}_m$	$\epsilon {\hat{R}}$	$\epsilon {\hat{A}}_m$	$\epsilon {\hat{A}}$	$\epsilon ^{\frac{1}{4}}{\bar{C}}_m$	$\epsilon ^{\frac{1}{2}}{\bar{C}}$	$\epsilon {\hat{B}}_m$	$\epsilon {\hat{B}}$	$\epsilon ^{\frac{1}{4}}{\bar{S}}_m$	$\epsilon {\hat{S}}$	$\epsilon ^{\frac{1}{4}}{\bar{E}}_m$	$\epsilon {\hat{E}}$	$\epsilon ^{\frac{1}{4}}{\bar{F}}_m$	$\epsilon ^{\frac{1}{2}}{\bar{F}}$
4	$\epsilon ^{\frac{1}{6}}{\tilde{T}}$	$\epsilon ^{\frac{1}{6}}{\tilde{R}}_m$	$\epsilon {\hat{R}}$	$\epsilon {\hat{A}}_m$	$\epsilon {\hat{A}}$	$\epsilon ^{\frac{1}{6}}{\tilde{C}}_m$	$\epsilon ^{\frac{1}{3}}{\tilde{C}}$	$\epsilon {\hat{B}}_m$	$\epsilon {\hat{B}}$	$\epsilon ^{\frac{1}{6}}{\tilde{S}}_m$	$\epsilon ^{\frac{5}{6}}{\tilde{S}}$	$\epsilon ^{\frac{1}{6}}{\tilde{E}}_m$	$\epsilon ^{\frac{5}{6}}{\tilde{E}}$	$\epsilon ^{\frac{1}{6}}{\tilde{F}}_m$	$\epsilon ^{\frac{1}{3}}{\tilde{F}}$
5	$\epsilon ^{\frac{1}{8}}T'$	$\epsilon ^{\frac{1}{8}}R'_m$	$\epsilon {\hat{R}}$	$\epsilon {\hat{A}}_m$	$\epsilon {\hat{A}}$	$\epsilon ^{\frac{1}{8}}C'_m$	$\epsilon ^{\frac{1}{4}}C'$	$\epsilon ^{\frac{7}{8}}B'_m$	$\epsilon {\hat{B}}$	$\epsilon ^{\frac{1}{8}}S'_m$	$\epsilon ^{\frac{3}{4}}S'$	$\epsilon ^{\frac{1}{8}}E'_m$	$\epsilon ^{\frac{3}{4}}E'$	$\epsilon ^{\frac{1}{8}}F'_m$	$\epsilon ^{\frac{1}{4}}F'$
6	$T^\dagger$	$R^\dagger _m$	$\epsilon {\hat{R}}$	$\epsilon {\hat{A}}_m$	$\epsilon {\hat{A}}$	$C^\dagger _m$	$C^\dagger$	$\epsilon ^{\frac{1}{2}}B^\dagger _m$	$\epsilon ^{\frac{1}{2}}B^\dagger$	$S^\dagger _m$	$\epsilon ^{\frac{1}{2}}S^\dagger$	$E^\dagger _m$	$\epsilon ^{\frac{1}{2}}E^\dagger$	$F^\dagger _m$	$F^\dagger$
7	$\frac{1}{2\kappa }\phi ^{-1}+T^\ddagger$	$R^\dagger _m$	$\epsilon {\hat{R}}$	$\epsilon ^{\frac{1}{2}}A^\ddagger _m$	$\epsilon ^{\frac{1}{2}}A^\ddagger$	$C^\dagger _m$	$\phi ^{-1}C^\ddagger$	$\epsilon ^{\frac{1}{2}}B^\dagger _m$	$\epsilon ^{\frac{1}{2}}\phi ^{-1}B^\ddagger$	$S^\dagger _m$	$\epsilon ^{\frac{1}{2}}\phi ^{-1}S^\ddagger$	$E^\dagger _m$	$\epsilon ^{\frac{1}{2}}\phi ^{-1}E^\ddagger$	$F^\dagger _m$	$\phi ^{-1}F^\ddagger$
8	$\frac{1}{\kappa }\phi ^{-1}+T^\diamond$	$R^\dagger _m$	$\epsilon {\hat{R}}$	$A^\diamond _m$	$A^\diamond$	$C^\dagger _m$	$\phi ^{-2}C^\diamond$	$\phi B^\diamond _m$	$B^\diamond$	$S^\dagger _m$	$\epsilon ^{\frac{1}{2}}\phi ^{-2} S^\diamond$	$E^\dagger _m$	$\epsilon ^{\frac{1}{2}}\phi ^{-2} E^\diamond$	$\epsilon ^{-\frac{1}{2}}\phi F^\diamond _m$	$\phi ^{-2} F^\diamond$
9	$\epsilon ^{-\frac{1}{2}}\left( \frac{1}{\kappa }\phi ^{-1}+T^+\right)$	$R^\dagger _m$	$\epsilon {\hat{R}}$	$A^\diamond _m$	$\epsilon ^{-\frac{1}{2}}A^+$	$\epsilon ^{-\frac{1}{2}}C^+_m$	$\phi ^{-3}C^+$	$\phi ^2B^+_m$	$\epsilon ^{-\frac{1}{2}}\phi B^+$	$S^\dagger _m$	$\phi ^{-2}S^+$	$E^\dagger _m$	$\phi ^{-2}E^+$	$\epsilon ^{-\frac{1}{2}}\phi F^\diamond _m$	$\phi ^{-4} F^+$
10	$\epsilon ^{-1}\left( \frac{1}{\kappa }\phi ^{-1}+\breve{T}\right)$	$R^\dagger _m$	$\epsilon {\hat{R}}$	$A^\diamond _m$	$\epsilon ^{-\frac{1}{2}}A^+$	$\epsilon ^{-\frac{1}{2}}C^+_m$	$\epsilon ^{-\frac{1}{2}}\phi ^{-3}\breve{C}$	$\epsilon ^{\frac{1}{2}}\phi ^2 \breve{B}_m$	$\epsilon ^{-\frac{1}{2}}\breve{B}$	$S^\dagger _m$	$\epsilon ^{-\frac{1}{2}} \phi ^{-2}\breve{S}$	$E^\dagger _m$	$\epsilon ^{-\frac{1}{2}} \phi ^{-2}\breve{E}$	$\phi \breve{F}_m$	$\phi ^{-4}\delta ^{-1}\breve{F}$

On this timescale, mRNA degradation and expression of *ramA* appear at leading order. Our system of equations rescaled for the sixth timescale is

$$\begin{aligned} {\frac{{ dR_m^\dagger }}{{ dT^\dagger }}}&=1-R_m^\dagger , \\ {\frac{{ d{\hat{A}}}_{{m}}}{{ dT^\dagger }}}&={\frac{{\hat{A}}}{ (\epsilon {\hat{A}}+1) (\epsilon {\hat{R}}+1) }}+\alpha -{\hat{A}}_{{m}}, \\ {\frac{{ dC_m^\dagger }}{{ dT^\dagger }}}&=\frac{\lambda }{\epsilon {\hat{A}}+\lambda }-C_m^\dagger , \\ {\frac{{ dB_m^\dagger }}{{ dT^\dagger }}}&={\frac{\epsilon ^\frac{1}{2}{\hat{A}}+S^\dagger }{ ( 1+\epsilon ^\frac{1}{2} S^\dagger +\epsilon {\hat{A}} ) ( 1+\epsilon ^\frac{1}{2} E^\dagger +C^\dagger ) }}-B_m^\dagger , \\ {\frac{{ dS_m^\dagger }}{{ dT^\dagger }}}&=1-S_m^\dagger , \\ {\frac{{ dE_m^\dagger }}{{ dT^\dagger }}}&=1-E_m^\dagger , \\ {\frac{{ dF_m^\dagger }}{{ dT^\dagger }}}&={\frac{{ \eta }}{{ \eta }+\epsilon ^\frac{1}{2} E^\dagger }}- F_m^\dagger ,\\ \epsilon {\frac{{ d{\hat{R}}}}{{ dT^\dagger }}}&= 0, \\ {\frac{{ d{\hat{A}}}}{{ dT^\dagger }}}&=\theta \, {\hat{A}}_{{m}}-\epsilon ^{\frac{1}{2}}\upsilon \, {\hat{A}}-\epsilon \varDelta {\hat{A}}, \\ {\frac{{ dC^\dagger }}{{ dT^\dagger }}}&= \gamma \, C_m^\dagger -\epsilon \varDelta C^\dagger , \\ {\frac{{ dB^\dagger }}{{ dT^\dagger }}}&=\beta \, B_m^\dagger -\epsilon \varDelta \,B^\dagger , \\ {\frac{{ dS^\dagger }}{{ dT^\dagger }}}&=\sigma \, S_m^\dagger -\epsilon \varDelta \,S^\dagger , \\ {\frac{{ dE^\dagger }}{{ dT^\dagger }}}&=\xi \, E_m^\dagger -\epsilon \varDelta \,E^\dagger , \\ {\frac{{ dF^\dagger }}{{ dT^\dagger }}}&={\frac{ F_m^\dagger }{\epsilon ^\frac{1}{2} \omega B^\dagger +1}}-\epsilon \varDelta F^\dagger . \end{aligned}$$

Solving the system of leading order equations and matching to the long term dominant behaviour on the previous timescale gives the following asymptotic approximations

48 $$\begin{aligned}&\displaystyle R_m^\dagger =1-e^{-T^\dagger },\qquad {\hat{R}}=R_0,\qquad C_m^\dagger =1-e^{-T^\dagger }, \nonumber \\&\displaystyle C^\dagger =\gamma \left( T^\dagger -e^{-T^\dagger } -1\right) ,\qquad B_m^\dagger =\frac{\sigma T^\dagger }{\gamma T^\dagger +1},\qquad S_m^\dagger =1-e^{-T^\dagger }, \nonumber \\&\displaystyle B^\dagger ={\frac{\beta \sigma }{\gamma }}\left( 1-{\frac{\ln \left( T^\dagger \gamma + 1 \right) }{{\gamma }}}\right) ,\qquad S^\dagger =\sigma \left( T^\dagger -e^{-T^\dagger } -1\right) ,\qquad E_m^\dagger =1-e^{-T^\dagger }, \nonumber \\&\displaystyle E^\dagger =\xi \left( T^\dagger -e^{-T^\dagger } -1\right) ,\qquad F^\dagger =T^\dagger -e^{-T^\dagger } -1,\qquad F_m^\dagger =1-e^{-T^\dagger }, \nonumber \\&\displaystyle {\hat{A}}_m={\frac{ \left( \sqrt{1+4\,\theta }\alpha +\sqrt{1+4\,\theta }A_{{0}}+2\,A_{{0} }\theta +\alpha +A_{{0}} \right) {{e}^{ \frac{\left( -1+\sqrt{1+4\,\theta } \right) T^\dagger }{2}}}}{2\sqrt{1+4\,\theta }}}-\alpha , \nonumber \\&\displaystyle {\hat{A}}={\frac{ \left( \left( \alpha +A_{{0}} \right) \sqrt{1+4\,\theta }+ \left( 2\,\theta +1 \right) A_{{0}}+\alpha \right) {{e}^{ \frac{\left( -1+\sqrt{1+4\,\theta } \right) T^\dagger }{2}}}}{2\sqrt{1+4\,\theta }}}. \end{aligned}$$

**Fig. 11:**
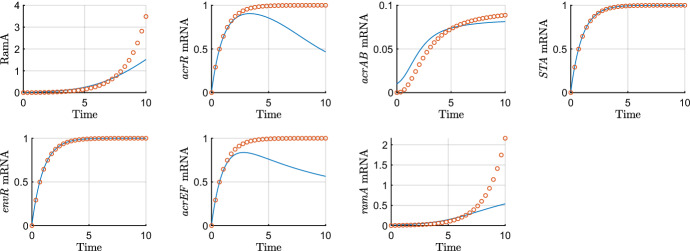
Asymptotic approximations on timescale 6 ($\epsilon =0.01$) for the mutant case ($m=0$). On this timescale, time is *O*(1), so we expect the asymptotics to be accurate around $T=1$

We plot asymptotic approximations of those variables that evolve on this timescale against the numerical solutions in Fig. 11. In this timescale, we have most of the mRNAs’ asymptotic approximations reaching near steady state as their degradation takes effect. The only mRNA not reaching steady state is *ramA* mRNA, which is exhibiting exponential growth. There is the same exponential growth for RamA protein, both of these growth behaviours are caused by the positive feedback loop with the *ramA* gene upon itself. In contrast to the wild-type system, without any presence of RamR protein to repress this feedback loop, we see rapidly increasing expression of *ramA*.

#### Timescale 7: activation of *acrAB* by RamA

For this timescale, we require the following values $\phi =\ln (1/\epsilon )^{-1}$ and $\kappa =\frac{1}{2}(-1+\sqrt{1+4\theta })$, resulting from the logarithmic and exponential behaviour on the previous timescale respectively. Activation of *acrAB* mRNA transcription via RamA protein now appears and our system of equations rescaled for the seventh timescale is

$$\begin{aligned} {\frac{{ dR_m^\dagger }}{{ dT^\ddagger }}}&=1-R_m^\dagger , \\ {\frac{dA_m^\ddagger }{{ dT^\ddagger }}}&={\frac{A^\ddagger }{ (\epsilon ^{\frac{1}{2}} A^\ddagger +1) (\epsilon {\hat{R}}+1) }}+\epsilon ^\frac{1}{2}\alpha -A_m^\ddagger , \\ {\frac{{ dC_m^\dagger }}{{ dT^\ddagger }}}&=\frac{\lambda }{\epsilon ^{\frac{1}{2}}A^\ddagger +\lambda }-C_m^\dagger , \\ {\frac{{ dB_m^\dagger }}{{ dT^\ddagger }}}&={\frac{A^\ddagger +\phi ^{-1} S^\ddagger }{ ( 1+\epsilon ^\frac{1}{2} \phi ^{-1} S^\ddagger +\epsilon ^{\frac{1}{2}} A^\ddagger ) ( 1+\epsilon ^\frac{1}{2} \phi ^{-1} E^\ddagger + \phi ^{-1} C^\ddagger ) }}-B_m^\dagger , \\ {\frac{{ dS_m^\dagger }}{{ dT^\ddagger }}}&=1-S_m^\dagger , \\ {\frac{{ dE_m^\dagger }}{{ dT^\ddagger }}}&=1-E_m^\dagger , \\ {\frac{{ dF_m^\dagger }}{{ dT^\ddagger }}}&={\frac{{ \eta }}{{ \eta }+\epsilon ^\frac{1}{2} \phi ^{-1} E^\ddagger }}- F_m^\dagger ,\\ \epsilon {\frac{{ d{\hat{R}}}}{{ dT^\ddagger }}}&= 0, \\ {\frac{{ dA^\ddagger }}{{ dT^\ddagger }}}&=\theta \, A_m^\ddagger -\epsilon ^\frac{1}{2}\upsilon \, A^\ddagger -\epsilon \varDelta A^\ddagger , \\ \phi ^{-1}{\frac{{ dC^\ddagger }}{{ dT^\ddagger }}}&= \gamma \, C_m^\dagger -\epsilon \phi ^{-1}\varDelta C^\ddagger , \\ \phi ^{-1}{\frac{{ dB^\ddagger }}{{ dT^\ddagger }}}&= \beta \, B_m^\dagger -\epsilon \phi ^{-1}\varDelta \,B^\ddagger , \\ \phi ^{-1}{\frac{{ dS^\ddagger }}{{ dT^\ddagger }}}&=\sigma \, S_m^\dagger -\epsilon \phi ^{-1}\varDelta \,S^\ddagger , \\ \phi ^{-1}{\frac{{ dE^\ddagger }}{{ dT^\ddagger }}}&=\xi \, E_m^\dagger -\epsilon \phi ^{-1}\varDelta \,E^\ddagger , \\ \phi ^{-1}{\frac{{ dF^\ddagger }}{{ dT^\ddagger }}}&={\frac{ F_m^\dagger }{\epsilon ^\frac{1}{2}\phi ^{-1} \omega B^\ddagger +1}}-\epsilon \phi ^{-1}\varDelta F^\ddagger . \end{aligned}$$

We solve the leading order system of ODEs and match to the long term dominant behaviour on the previous timescale, giving us the following asymptotic approximations

49 $$\begin{aligned} R_m^\dagger&=1-e^{-T^\ddagger },\qquad {\hat{R}}=R_0,\qquad C_m^\dagger =1-e^{-T^\ddagger } \nonumber \\ C^\ddagger&=\phi \gamma \left( T^\ddagger -e^{-T^\ddagger } -1\right) ,\qquad B_m^\dagger =\frac{A_0e^{\theta T^\ddagger }+\phi ^{-1}\sigma T^\ddagger }{\phi ^{-1}\gamma T^\ddagger +1},\qquad S_m^\dagger =1-e^{-T^\ddagger }, \nonumber \\ S^\ddagger&=\phi \sigma \left( T^\ddagger -e^{-T^\ddagger } -1\right) ,\qquad E_m^\dagger =1-e^{-T^\ddagger },\qquad F_m^\dagger =1-e^{-T^\ddagger }, \nonumber \\ E^\ddagger&=\phi \xi \left( T^\ddagger -e^{-T^\ddagger } -1\right) ,\qquad F^\ddagger =\phi (T^\ddagger -e^{-T^\ddagger } -1),\nonumber \\ A^\ddagger _m&={\frac{ \left( A_{{0}}\sqrt{1+4\,\theta }+2\,A_{{{ m0}}} \theta +A_{{0}} \right) \left( -1+\sqrt{1+4\,\theta } \right) {{e} ^{\frac{ \left( -1+\sqrt{1+4\,\theta } \right) T^\ddagger }{2}}}}{4\theta \sqrt{1+4\,\theta } }}, \nonumber \\ A^\ddagger&={\frac{ \left( A_{{0}}\sqrt{1+4\,\theta }+2\,A_{{{ m0}}} \theta +A_{{0}} \right) {{e}^{\frac{\left( -1+\sqrt{1+4\,\theta } \right) T^\ddagger }{2}}}}{2\sqrt{1+4\,\theta }}}, \nonumber \\ B^\ddagger&={\frac{\beta \,\phi \,A_{{0}}}{\gamma }{\mathrm{e}^{-{\frac{\theta }{\gamma }}}}{ Ei} \left( 1,\theta \,T+{\frac{\theta }{\gamma }} \right) }+{ \frac{\beta \,\phi \,\sigma \,T}{\gamma }}-{\frac{\beta \,\phi \,\sigma \, \ln \left( T\gamma \,\phi \,\theta +\phi \,\theta \right) }{{\gamma }^{2}}}. \end{aligned}$$

**Fig. 12:**
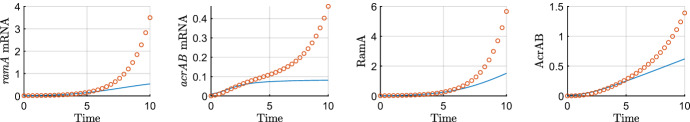
Asymptotic approximations on timescale 7 ($\epsilon =0.01$). On this timescale, time is $O(1+\frac{1}{2\kappa }\phi ^{-1})$, so we expect the asymptotics to be accurate around $T=1+\frac{1}{2\kappa }\phi ^{-1}\approx 4.7257$

We plot asymptotic approximations of those variables that evolve on this timescale against the numerical solutions in Fig. 12. In this timescale we still have exponential growth of *ramA* mRNA and RamA protein caused by the positive feedback of the *ramA* gene. This growth has resulted in activation of *acrAB* mRNA which now exhibits long term exponential growth. This is being translated to AcrAB protein which now also exhibits long term exponential growth.

#### Timescale 8: *ramA* mRNA reaching steady state

For this timescale, the limitation of *ramA* activating its own expression enters the leading order balance, our system of equations rescaled for the eighth timescale is

$$\begin{aligned} {\frac{{ dR_m^\dagger }}{{ dT^\diamond }}}&=1-R_m^\dagger , \\ {\frac{dA_m^\diamond }{{ dT^\diamond }}}&={\frac{A^\diamond }{ (A^\diamond +1) (\epsilon {\hat{R}}+1) }}+\epsilon \alpha -A_m^\diamond , \\ {\frac{{ dC_m^\dagger }}{{ dT^\diamond }}}&=\frac{\lambda }{A^\diamond +\lambda }-C_m^\dagger , \\ \phi {\frac{{ dB_m^\diamond }}{{ dT^\diamond }}}&={\frac{A^\diamond +\epsilon ^\frac{1}{2} \phi ^{-2} S^\diamond }{ ( 1+\epsilon ^\frac{1}{2} \phi ^{-2} S^\diamond + A^\diamond ) ( 1+\epsilon ^\frac{1}{2} \phi ^{-2} E^\diamond + \phi ^{-2} C^\diamond ) }}-\phi B_m^\diamond , \\ {\frac{{ dS_m^\dagger }}{{ dT^\diamond }}}&=1-S_m^\dagger , \\ {\frac{{ dE_m^\dagger }}{{ dT^\diamond }}}&=1-E_m^\dagger , \\ {\frac{{ dF_m^\dagger }}{{ dT^\diamond }}}&={\frac{{ \eta }}{{ \eta }+\epsilon ^\frac{1}{2} \phi ^{-2} E^\diamond }}- F_m^\dagger ,\\ \epsilon {\frac{{ d{\hat{R}}}}{{ dT^\diamond }}}&= 0, \\ {\frac{{ dA^\diamond }}{{ dT^\diamond }}}&=\theta \, A_m^\diamond -\epsilon ^\frac{1}{2}\upsilon \, A^\diamond -\epsilon \varDelta A^\diamond , \\ \phi ^{-2}{\frac{{ dC^\diamond }}{{ dT^\diamond }}}&= \gamma \, C_m^\dagger -\epsilon \phi ^{-2}\varDelta C^\diamond , \\ {\frac{{ dB^\diamond }}{{ dT^\diamond }}}&=\phi \beta \, B_m^\diamond -\epsilon \varDelta \,B^\diamond , \\ \phi ^{-2}{\frac{{ dS^\diamond }}{{ dT^\diamond }}}&=\sigma \, S_m^\dagger -\epsilon \phi ^{-2}\varDelta \,S^\diamond , \\ \phi ^{-2}{\frac{{ dE^\diamond }}{{ dT^\diamond }}}&=\xi \, E_m^\dagger -\epsilon \phi ^{-2}\varDelta \,E^\diamond , \\ \phi ^{-2}{\frac{{ dF^\diamond }}{{ dT^\diamond }}}&={\frac{ F_m^\dagger }{\omega B^\diamond +1}}-\epsilon \phi ^{-2}\varDelta F^\diamond . \end{aligned}$$

Finding the leading order balance, solving and matching to the long term dominant behaviour on the previous timescale gives the following asymptotic approximations.

50 $$\begin{aligned} R_m^\dagger&=1-e^{-T^\diamond },\qquad {\hat{R}}=R_0,\qquad C_m^\dagger =1-e^{-T^\diamond }, \nonumber \\ C^\diamond&={\frac{{\phi }^{2}\gamma \,\lambda \,\ln \left( \theta \,T^\diamond +\lambda \right) }{\theta }},\qquad F_m^\dagger =1-e^{-T^\diamond },\qquad S_m^\dagger =1-e^{-T^\diamond }, \nonumber \\ S^\diamond&=\phi ^2\sigma \left( T^\diamond -e^{-T^\diamond } -1\right) ,\qquad E_m^\dagger =1-e^{-T^\diamond },\qquad E^\diamond =\phi ^2\xi \left( T^\diamond -e^{-T^\diamond } -1\right) , \nonumber \\ A^\diamond _m&=\frac{\mathrm{W} \left( {\frac{ \left( A_{{0}}\sqrt{1+4\theta }+2A_{{{ m0}}}\theta +A_{{0}} \right) {{e}^{\theta T^\diamond }}}{2\sqrt{1+4 \theta }}}\right) }{\mathrm{W} \left( {\frac{ \left( A_{{0}}\sqrt{1+4\theta }+2A_{{{ m0}}}\theta +A_{{0}} \right) {{e}^{\theta T^\diamond }}}{2\sqrt{1+4 \theta }}}\right) +1}, \nonumber \\ A^\diamond&=\mathrm{W} \left( {\frac{ \left( A_{{0}}\sqrt{1+4\theta }+2A_{{{ m0}}}\theta +A_{{0}} \right) {{e}^{\theta T^\diamond }}}{2\sqrt{1+4 \theta }}}\right) , \nonumber \\ B_m^\diamond&=\frac{\phi ^{-1}\theta T^\diamond }{(1+\theta T^\diamond )(1+\frac{\gamma \,\lambda \,\ln \left( \theta \,T^\diamond +\lambda \right) }{\theta })}, \nonumber \\ B^\diamond&={\frac{\beta }{\gamma }{{e}^{-{\frac{\theta }{\gamma \lambda }}}}{ Ei} \left( 1,\ln (\theta T+\lambda )+{\frac{\theta }{\gamma \lambda }} \right) }-\frac{\beta }{\gamma }\ln \left( \gamma \lambda \ln (\theta T+\lambda )+\theta \right) , \nonumber \\ F^\diamond&=\frac{\phi ^2}{\beta \omega }\left( \frac{\gamma }{2\theta }\ln ^2(\theta T^\diamond +\lambda )+\frac{1}{\lambda }\ln (\theta T^\diamond +\lambda )\right) . \end{aligned}$$

**Fig. 13:**
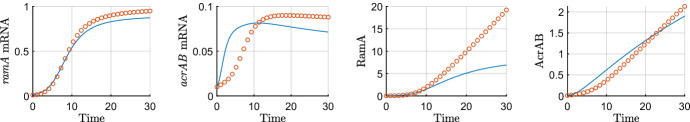
Asymptotic approximations on timescale 8 ($\epsilon =0.01$). On this timescale, time is $O(1+\frac{1}{\kappa }\phi ^{-1})$, so we expect the asymptotics to be accurate around $T=1+\frac{1}{\kappa }\phi ^{-1}\approx 8.4513$

We plot asymptotic approximations of those variables that evolve on this timescale against the numerical solutions in Fig. 13. In this timescale the *ramA* gene’s positive feedback has been limited, causing at long term the gene’s mRNA to approach steady state and the gene’s protein to have linear growth. This change of behaviour of RamA from exponential growth causes *acrAB* mRNA and protein to no longer display exponential behaviour.

#### Timescale 9: degradation of RamA protein, inhibition of *acrAB* and *acrEF*

For this timescale, degradation of RamA protein caused by Lon Protease enters the leading order balance as well as EnvR repression of *acrEF*, our system of equations rescaled for the ninth timescale is

$$\begin{aligned} \epsilon ^{\frac{1}{2}}{\frac{{ dR_m^\dagger }}{{ dT^+}}}&=1-R_m^\dagger , \\ \epsilon ^{\frac{1}{2}}{\frac{dA_m^\diamond }{{ dT^+}}}&={\frac{\epsilon ^{-\frac{1}{2}}A^+}{ (\epsilon ^{-\frac{1}{2}}A^++1) (\epsilon {\hat{R}}+1) }}+\epsilon \alpha -A_m^\diamond , \\ \epsilon {\frac{{ dC_m^+}}{{ dT^+}}}&=\frac{\lambda }{\epsilon ^{-\frac{1}{2}}A^++\lambda }-\epsilon ^{\frac{1}{2}}C_m^+, \\ \epsilon ^{\frac{1}{2}}\phi ^2{\frac{{ dB_m^+}}{{ dT^+}}}&={\frac{\epsilon ^{-\frac{1}{2}}A^++\phi ^{-2} S^+}{ ( 1+\phi ^{-2} S^++ \epsilon ^{-\frac{1}{2}}A^+ ) ( 1+\phi ^{-2} E^++ \phi ^{-3} C^+ ) }}-\phi ^2 B_m^+, \\ \epsilon ^{\frac{1}{2}}{\frac{{ dS_m^\dagger }}{{ dT^+}}}&=1-S_m^\dagger , \\ \epsilon ^{\frac{1}{2}}{\frac{{ dE_m^\dagger }}{{ dT^+}}}&=1-E_m^\dagger , \\ \epsilon ^{\frac{1}{2}}{\frac{{ dF_m^\dagger }}{{ dT^+}}}&={\frac{{ \eta }}{{ \eta }+ \phi ^{-2} E^+}}- F_m^\dagger ,\\ \epsilon ^{\frac{3}{2}}{\frac{{ d{\hat{R}}}}{{ dT^+}}}&= 0, \\ {\frac{{ dA^+}}{{ dT^+}}}&=\theta \, A_m^\diamond -\upsilon \, A^+-\epsilon ^{\frac{1}{2}}\varDelta A^+, \\ \phi ^{-3}{\frac{{ dC^+}}{{ dT^+}}}&= \gamma \, C_m^+-\epsilon ^\frac{1}{2}\phi ^{-3}\varDelta C^+, \\ {\frac{{ dB^+}}{{ dT^+}}}&=\phi \beta \, B_m^+-\epsilon ^{\frac{1}{2}}\varDelta \,B^+, \\ \phi ^{-2}{\frac{{ dS^+}}{{ dT^+}}}&=\sigma \, S_m^\dagger -\epsilon ^\frac{1}{2}\phi ^{-2}\varDelta \,S^+, \\ \phi ^{-2}{\frac{{ dE^+}}{{ dT^+}}}&=\xi \, E_m^\dagger -\epsilon ^\frac{1}{2}\phi ^{-2}\varDelta \,E^+, \\ \phi ^{-4}{\frac{{ dF^+}}{{ dT^+}}}&={\frac{F_m^\dagger }{\phi \omega B^++\epsilon ^\frac{1}{2}}}-\epsilon ^\frac{1}{2}\phi ^{-4}\varDelta F^+. \end{aligned}$$

Solving the leading order system of ODEs, matching to the long term dominant behaviour on the previous timescale gives the following asymptotic approximations

51 $$\begin{aligned} A^\diamond&=\frac{\theta }{\upsilon }(1-e^{-\upsilon T^+}),\qquad A^\diamond _m=1,\qquad R_m^\dagger =1, \nonumber \\ {\hat{R}}&=R_0,\qquad C_m^+=\frac{\lambda \upsilon }{\theta (1-e^{-\upsilon T^+})},\qquad C^+=\frac{\phi ^3\gamma \lambda \upsilon }{\theta }T^+, \nonumber \\ S_m^\dagger&=1,\qquad S^+=\phi ^2\sigma T^+,\qquad B_m^+=\frac{1}{\phi ^2(1+\xi T^++\frac{\gamma \lambda \upsilon }{\theta }T^+)}, \nonumber \\ E_m^\dagger&=1,\qquad E^+=\phi ^2\xi T^+,\qquad F_m^\dagger =\frac{\eta }{\eta +\xi T^+}, \nonumber \\ B^+&={\frac{\beta \,{\phi }^{2}\theta \,\ln \left( \left( {\phi }^{3}\gamma \,\lambda \,\upsilon +{\phi }^{3}\theta \,\xi \right) T^++{\phi }^{3}\theta \right) }{{\phi }^{3}\gamma \,\lambda \,\upsilon +{\phi }^{3}\theta \,\xi }}, \nonumber \\ F^+&=\frac{\phi ^4\eta (\gamma \lambda \upsilon +\theta \xi )}{\omega \xi \beta \theta }\ln (\phi ^3\ln (T^++1)). \end{aligned}$$

**Fig. 14:**
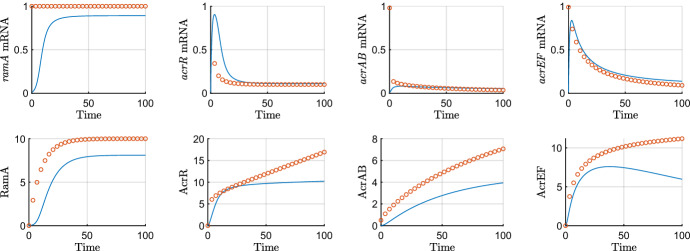
Figures showing asymptotic approximations using timescale 9 to the full solutions for $\epsilon =0.01$. Here the time scaling is $O(\epsilon ^{-\frac{1}{2}}(1+\frac{1}{\kappa }\phi ^{-1}))$, so we expect the asymptotics to be accurate around $T=\epsilon ^{-\frac{1}{2}}(1+\frac{1}{\kappa }\phi ^{-1})\approx 84.5132$. Discrepancies between the asymptotic approximations and numerical simulations could be reduced by using a smaller epsilon value

We plot asymptotic approximations of those variables that evolve on this timescale against the numerical solutions in Fig. 14. In this timescale we have RamA protein reaching steady state from degradation via Lon Protease. With this, we have *acrR* mRNA also reaching steady state. With less RamA protein due to degradation, the repressors dominate expression of *acrAB*, causing its inhibition. We also have EnvR protein causing inhibition of *acrEF* mRNA. This inhibition of mRNAs causes logarithmic behaviour for both AcrAB and AcrEF.

#### Timescale 10: full protein degradation, with all proteins reaching steady state

For this timescale, we use $\delta =\ln (\ln (1/\epsilon ))^{-1}$, emerging from the logarithmic behaviour on the previous timescale. On this timescale protein degradation emerges for the rest of our proteins. Our system of equations rescaled for the tenth timescale is

$$\begin{aligned} \epsilon {\frac{{ dR_m^\dagger }}{{ d\breve{T}}}}&=1-R_m^\dagger , \\ \epsilon {\frac{dA_m^\diamond }{{ d\breve{T}}}}&={\frac{\epsilon ^{-\frac{1}{2}}A^+}{ (\epsilon ^{-\frac{1}{2}}A^++1) (\epsilon {\hat{R}}+1) }}+\epsilon \alpha -A_m^\diamond , \\ \epsilon ^{\frac{3}{2}}{\frac{{ dC_m^+}}{{ d\breve{T}}}}&=\frac{\lambda }{\epsilon ^{-\frac{1}{2}}A^++\lambda }-\epsilon ^{\frac{1}{2}}C_m^+, \\ \epsilon ^{\frac{3}{2}}\phi ^2{\frac{{ d\breve{B}_m}}{{ d\breve{T}}}}&={\frac{\epsilon ^{-\frac{1}{2}}A^+ +\epsilon ^{-\frac{1}{2}}\phi ^{-2} \breve{S}}{ ( 1+\epsilon ^{-\frac{1}{2}}\phi ^{-2} \breve{S}+ \epsilon ^{-\frac{1}{2}}A^+ ) ( 1+\epsilon ^{-\frac{1}{2}}\phi ^{-2} \breve{E}+ \epsilon ^{-\frac{1}{2}}\phi ^{-3} \breve{C} ) }}-\epsilon ^{\frac{1}{2}}\phi ^2 \breve{B}_m, \\ \epsilon {\frac{{ dS_m^\dagger }}{{ d\breve{T}}}}&=1-S_m^\dagger , \\ \epsilon {\frac{{ dE_m^\dagger }}{{ d\breve{T}}}}&=1-E_m^\dagger , \\ \epsilon ^{\frac{3}{2}}{\frac{{ d\breve{F}_m}}{{ d\breve{T}}}}&={\frac{{ \eta }}{{ \eta }+ \epsilon ^{-\frac{1}{2}}\phi ^{-2} \breve{E}}}- \epsilon ^{\frac{1}{2}}\breve{F}_m,\\ \epsilon ^2{\frac{{ d{\hat{R}}}}{{ d\breve{T}}}}&= 0, \\ \epsilon ^{\frac{1}{2}}{\frac{{ dA^+}}{{ d\breve{T}}}}&=\theta \, A_m^\diamond -\upsilon \, A^+-\epsilon ^{\frac{1}{2}}\varDelta A^+, \\ \phi ^{-3}{\frac{{ d\breve{C}}}{{ d\breve{T}}}}&=\gamma \, C_m^+-\phi ^{-3}\varDelta \breve{C}, \\ {\frac{{ d\breve{B}}}{{ d\breve{T}}}}&=\phi ^2 \beta \, \breve{B}_m-\varDelta \,\breve{B}, \\ \phi ^{-2}{\frac{{ d\breve{S}}}{{ d\breve{T}}}}&=\sigma \, S_m^\dagger -\phi ^{-2}\varDelta \,\breve{S}, \\ \phi ^{-2}{\frac{{ d\breve{E}}}{{ d\breve{T}}}}&=\xi \, E_m^\dagger -\phi ^{-2}\varDelta \,\breve{E}, \\ \phi ^{-4}\delta ^{-1}{\frac{{ d\breve{F}}}{{ d\breve{T}}}}&={\frac{\breve{F}_m}{\phi \omega \breve{B}+\epsilon ^\frac{1}{2}}}-\phi ^{-4}\delta ^{-1}\varDelta \breve{F}. \end{aligned}$$

This system of ODEs can be solved, matching to the long term dominant behaviour on the previous timescale, giving the following asymptotic approximations.

52 $$\begin{aligned} R_m^\dagger&=1,&{\hat{R}}&=R_0,&A^+_m&=1, \nonumber \\ A^+&=\frac{\theta }{\upsilon },&\breve{C}&=\phi ^3\frac{\gamma \lambda \upsilon }{\varDelta \theta }(1-e^{-\varDelta \breve{T}}),&C_m^+&=\frac{\lambda \upsilon }{\theta }, \nonumber \\ \breve{B}_m&=\frac{1}{\phi ^2(\frac{\xi }{\varDelta }+\frac{\gamma \lambda \upsilon }{\varDelta \theta })(1-e^{-\varDelta \breve{T}})},&\breve{B}&=\frac{\beta \theta }{\gamma \lambda \upsilon +\theta \xi }(1-e^{-\varDelta \breve{T}}),&S_m^\dagger&=1, \nonumber \\ \breve{S}&=\phi ^2\frac{\sigma }{\varDelta }(1-e^{-\varDelta \breve{T}}),&\breve{E}&=\phi ^2\frac{\xi }{\varDelta }(1-e^{-\varDelta \breve{T}}),&E_m^\dagger&=1, \nonumber \\ \breve{F}_m&=\frac{\eta }{\frac{\xi }{\varDelta }(1-e^{-\varDelta \breve{T}})},&\breve{F}&=\frac{\phi ^{4}\delta \eta \left( \gamma \,\lambda \,\upsilon +\theta \xi \right) }{\xi \omega \beta \theta }(1-e^{-\varDelta \breve{T}}). \end{aligned}$$

**Fig. 15:**
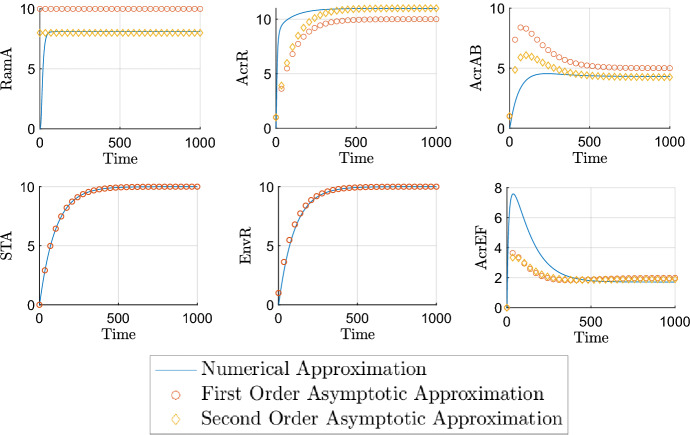
Asymptotic approximations on timescale 10 ($\epsilon =0.01$). On this timescale, time is $O(\epsilon ^{-1}(1+\frac{1}{\kappa }\phi ^{-1}))$, so we expect the asymptotics to be accurate around $T=\epsilon ^{-1}(1+\frac{1}{\kappa }\phi ^{-1}))\approx 845.1322$

We plot asymptotic approximations of those variables that evolve on this timescale against the numerical solutions in Fig. 15. In this timescale we have all variables reaching steady state. Due to disparity in the asymptotic approximations of some variables, we have included the second order terms in the asymptotic approximations (we could instead use a smaller value for epsilon).

### Steady state analysis

Upon reaching the final timescale, all of our variables attain a steady state. For the full nondimensionalised model it is not possible to derive a set of analytically solvable steady states. However, in both wild-type and mutant cases we can achieve achieve analytical expressions for the asymptotic approximation of the steady states. We know from our GRN that reducing the concentration of the main efflux pump protein AcrAB results in increased concentration of the homologue efflux pump protein AcrEF. Thus we must consider both efflux pump protein concentrations simultaneously. We perform a sensitivity analysis of the sum of the asymptotic approximations of the steady states of AcrAB (including second order terms) and AcrEF, the proteins that form the efflux pump complexes (ie this reflects the total efflux “power” of the bacteria). By conducting this analysis we hope to identify potential targets for efflux inhibition. Here, we use relative sensitivity in order to draw comparisons on how much individually changing a parameter affects the overall efflux. We define our equation for the relative sensitivity as

53 $$\begin{aligned} \varsigma =\frac{d ({\bar{B}}+{\bar{F}})}{d P}, \end{aligned}$$

where $d ({\bar{B}}+{\bar{F}})$ represents the change of the efflux pump genes steady state and *dP* represents the change of the nondimensional parameter being varied. We have performed numerical investigations into possible equilibria using the software XPPAUT. This uses Monte Carlo sampling on individual parameters and/or initial conditions and provides possible equilibria at these states. Whilst this is non-exhaustive, the results have given us no indication that there is ever more than one plausible (i.e. non-negative) steady state.

**Fig. 16:**
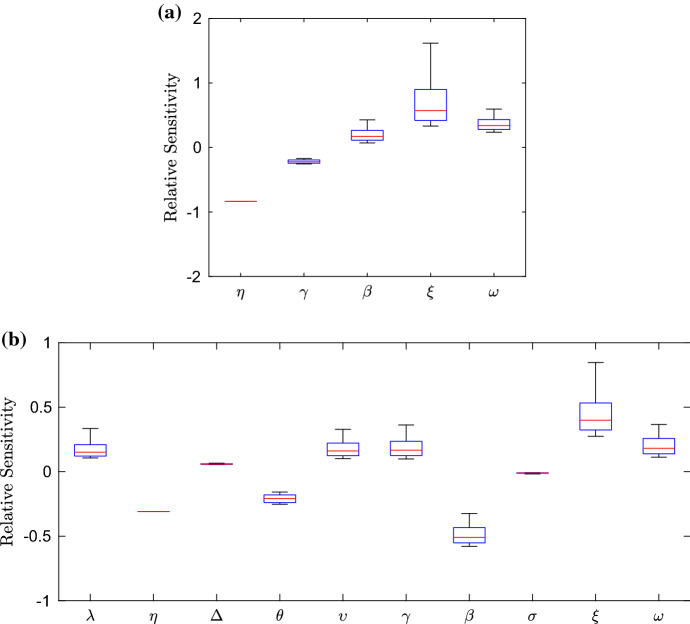
Boxplots showing the relative sensitivity of nondimensional parameters on the combined asymptotic approximated steady states of AcrAB and AcrEF. In (**a**) we denote the sensitivity in the wild-type case whereas in (**b**) we denote the mutant case. For (**b**), mutations to RamR protein results in more parameters involved in our steady state approximation

To conduct our sensitivity analysis, we vary all our nondimensional parameters in a bounded parameter space. For both wild-type and mutant strains, the space is bounded to the range of $\epsilon ^{\frac{1}{5}}$ to $\epsilon ^{-\frac{1}{5}}$ to maintain consistency with the parameter sizes used in the asymptotic analysis. By using a Latin hypercube method of sampling, we choose 10000 points in the parameter space for each parameter and find the relative sensitivity for each point. The resulting relative sensitivities are then plotted on box plots in order for us to view the distribution of sensitivity. We exhibit the results of the sensitivity analysis in Fig. 16.

We can see from the wild-type case (a), that the parameter to which efflux is most sensitive is $\xi$, which also has the largest spread of sensitivity of all parameters in this case. Our next most sensitive parameter is $\eta$, here all points correspond to the same value as this grouping only affects AcrEF and does so linearly. We note that our most sensitive parameter groupings $\xi$ and $\eta$ relate to the binding coefficients of EnvR to the two efflux pump genes, and the expression of *envR* respectively. Since both of these parameters involve *envR* mRNA or protein, the analysis suggests that this gene could be a possible target for inhibition of efflux in this case. Our next most sensitive parameter is $\omega$ relating to the link between the concentration of AcrAB and activation/repression of *acrEF* the homologue efflux pump gene. Unfortunately as we do not know the full mechanisms involved causing this link, this does not provide a realistic target for inhibition. However, this does lead us to believe that with more biological knowledge of this link there could be a potential inhibition target worth pursuing. Finally with similar sensitivities are $\gamma$ (*acrR* expression) and $\beta$ (*acrAB* expression). Since the former of these parameters has a relatively low sensitivity compared to other parameters, the analysis predicts that this may not be a target worth pursuing. The latter is an expected target, relating to direct expression of one of the efflux pump genes. It is interesting to note that some parameters in the system (that do not affect the efflux pump genes directly) provide a greater sensitivity than $\beta$, that is directly related to AcrAB concentration.

In the mutant case (b), it comes to note that we have double the amount of parameters that affect the efflux pump steady states compared to the wild-type case. This is partially due to including second order terms, however it is only the parameters $\varDelta$ and $\sigma$ that do not appear at leading order. With only one change in the GRN (to RamR protein), the change in the amount of parameters demonstrates the unpredictability and sensitive nature of this network. We note that here, the parameter to which the steady state of efflux proteins is most sensitive is $\xi$ (*envR* expression). Additionally we also see high sensitivity to the parameter $\eta$ (EnvR binding affinity). This similarity with the wild-type system further highlights the case for targeting the gene *envR* for inhibiting efflux. Our next most sensitive parameter is $\beta$ (*acrAB* expression) which differs from the wild-type case where it was one of the least sensitive parameters. This could be due to the overexpression of *acrAB* in this mutant case. The parameters $\lambda$ (RamA binding affinity), $\theta$ (*ramA* expression), $\upsilon$ (RamA degradation from Lon Protease), $\gamma$ (*acrR* expression) and $\omega$ (AcrAB and *acrEF* link) all show a degree of sensitivity, meaning that any of these parameters could prove to be a realistic target to inhibit efflux. However it is interesting to note that parameters associated with *ramA*, which is over expressed in this mutant case, is not the most sensitive target for inhibiting efflux. The rest of the parameters $\varDelta$ (degradation of mRNA and proteins) and $\sigma$ (secondary TA mRNA expression) have a low sensitivity in this case, which we should expect as these parameters are only prevalent in the second order terms. Thus the analysis suggests that these parameters may not be realistic targets for inhibiting efflux.

## Discussion

**Fig. 17:**
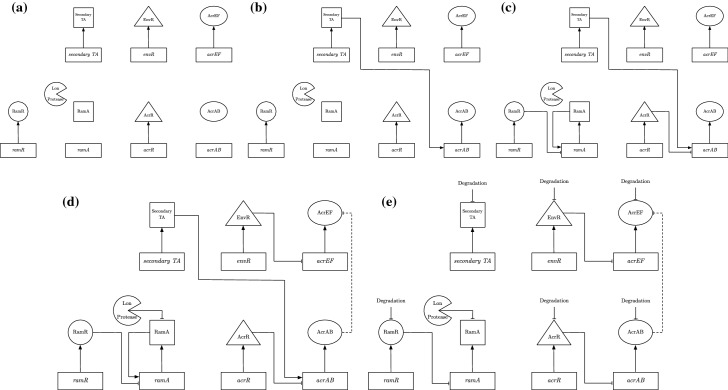
Schematic diagrams showing the order in which processes appear at leading order in the asymptotic analysis for the wild-type case. We show these processes in timescale order, starting from initial processes and finishing at steady state in (**e**). Here the dashed line represents the indirect link between AcrAB and AcrEF protein levels. As we evolve the schematic, we insert and remove arrows or lines depending on which terms enter or leave the leading order balance

Antimicrobial resistance is a topic with ever increasing importance. With the threat to human health worsening as more bacteria evolve resistance to antibiotics, it is clear we must urgently seek novel treatment methods in order to combat antibiotic resistance. By delving into GRNs governing resistance mechanisms, it is possible to identify certain targets to potentially prevent resistance in bacteria. We believe that our asymptotic analysis has given us useful insights into the network governing efflux pump expression. In Fig. 17 we exhibit the leading order processes in timescale order from our asymptotic analysis for the wild-type case. We detail the order of dominant processes shown in the schematics. As predicted by the mathematical analysis:

- Genes that are not highly regulated by proteins are expressed, resulting in their mRNA transcription and protein translation.
- If produced subject to the relevant stress, the secondary TAs (SoxS, MarA and Rob) do not significantly increase *acrAB* expression but the asymptotic analysis reveals that they may effect the timescale on which expression of *acrAB* first occurs.
- When produced, RamR inhibits *ramA* expression, preventing RamA from achieving activation of *acrAB* at leading order. AcrR also lowers (but does not shut off entirely) transcription of *acrAB*.
- EnvR binds to the promoter site of *acrEF* repressing its transcription.
- Degradation of all proteins brings the system to a mathematical steady state. The system would remain at this state with efflux proteins present until the relevant stress is removed from the cells, at which point the system would revert to a state of basal efflux.

We note that at steady state, the local repressors of the efflux pumps have been expressed to a large enough concentration that they are dominant in the leading order processes and are the only gene products impacting efflux pump expression. We can see at this point the system is reduced to four genes (*acrR*, *envR*, *acrAB* and *acrEF*) affecting efflux pump expression, and thus at steady state for this case we should focus on these genes as potential inhibition targets.

**Fig. 18:**
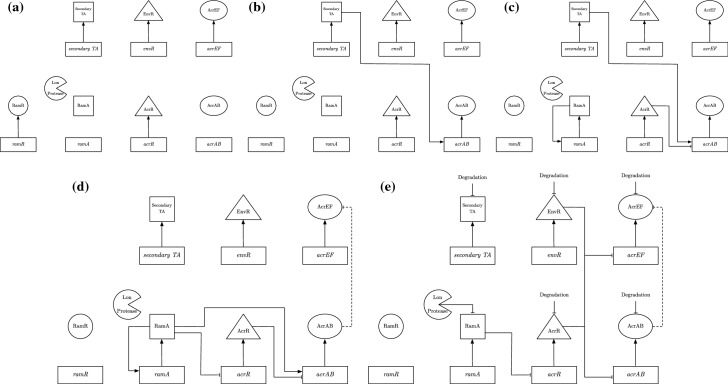
Schematic diagrams showing the order in which processes appear at leading order in the asymptotic analysis for the mutant case. We show these processes in timescale order, starting from initial processes and finishing at steady state in (**e**). Here the dashed line represents the indirect link between AcrAB and AcrEF protein levels. As we evolve the schematic, we insert and remove arrows or lines depending on which terms enter or leave the leading order balance

In Fig. 18 we exhibit the leading order processes in timescale order from our asymptotic analysis for the mutant case. We note that the schematics are not on identical timescales to the wild-type case as the scalings to reach each of the timescales are different. Here we detail the differences in the order of dominant processes shown in the schematics compared to the wild-type case. As predicted by the mathematical analysis:

- In this case, functional RamR is not produced. This allows the positive feedback loop on *ramA* expression to dominate at leading order, resulting in high production of RamA and activation of *acrAB* expression. Any activation by secondary TAs (that may occur under the appropriate stress) is overshadowed by RamA and relegated to lower order behaviour. RamA also lowers AcrR levels, yielding higher expression of *acrAB* in the analysis.
- RamA is regulated by degradation through the Lon protease, allowing AcrR and EnvR to dominate mathematically *acrAB* expression.
- Degradation of all proteins brings the system to a mathematical steady state.

At steady state, as expected this mutant strain also has RamA dominating the behaviour (in addition to those considered in the wild type strain). The analysis therefore identifies *ramA*, *acrR*, *envR*, *acrAB* and *acrEF* as the most likely potential targets for efflux pump inhibition. In regards to the other timescales, this breakdown highlights the importance of the positive feedback loop of *ramA*. With the release of *ramA* expression in this mutant case, various different interactions between genes and proteins become dominant. In particular, we see direct and indirect activation of *acrAB*, with the latter as a result of its local repressor, *acrR*, itself being inhibited (by RamA). It is interesting to note that mathematically the direct activation from RamA only dominates at leading order prior to steady state and without undergoing our time-dependent analysis we may have not identified this key mechanism in the activation of the efflux pump genes under this parameter set. Whilst at steady state other genes may prove to be more likely targets for inhibition (shown in Sect. 4.3), reducing the activation from RamA may be enough to minimise early expression of *acrAB*, enabling the antibiotic to kill bacteria before its efflux pumps are overexpressed. For example, an efflux inhibiting adjuvant targeting RamA may be most successful if administered with or before antibiotic.

It is also important to note that in the wild-type case we see no leading order activation processes caused by RamA (though this will be present at lower orders). Thus whilst choosing *ramA* as an inhibition target seems plausible, this may only revert the GRN to the wild-type case rather than knocking out efflux expression entirely.

## Summary

With the ever growing threat of antibiotic resistance, MDR *Salmonella* have been listed as a high priority for which new treatment methods are required (WHO 2017). One of the main defensive mechanisms used by Salmonella is efflux pumps that can expel multiple different antibiotics from the cytoplasm of the cell. The AcrAB-TolC and the AcrEF-TolC systems have been identified as major efflux pumps that contribute to MDR (Blair et al. 2014b). Inhibition of these efflux pump systems is a potential method to combat antibiotic resistance in bacteria, preventing the bacteria from being able to expel antibiotics via active transport (Piddock 2006). However, inhibition of these efflux pumps is a complex process, as the regulation of these efflux pumps are governed by complex gene regulation networks and inhibition of one efflux pump system can cause up regulation of another efflux pump system. These GRNs contain multiple different genes and proteins that interact with each other’s expression, ultimately leading to the expression of the genes that produce structural efflux pump proteins when the cell is under stress. The genes within these networks vary in expression between different strains, with over-expression of efflux pump genes being common in mutant MDR strains (Webber and Piddock 2003). In this paper we consider two strains, a wild-type strain and a mutant strain. Both of these strains consist of the same genes governing efflux pump expression, however the latter has nonfunctional RamR protein which indirectly causes over-expression of efflux pump genes. Thus in order for an inhibition adjuvant to antibiotic treatment to be developed, the GRN processes must first be fully understood. For the inhibition to be effective, it must be able to repress the efflux pump systems in multiple different strains.

We have used various mathematical modelling techniques in order to greater understand the processes controlling efflux pump expression. We have developed an ODE model, of which most parameters are not currently available from experimental data. Thus we have applied asymptotic techniques to reduce the need for specific parameter values. We first nondimensionalised our model, resulting in nondimensional parameter groupings. By using information from the biology of the network, we were able to estimate sizes for these parameter groupings and focus on relative parameter sizes rather than absolute parameter values.

This approach enabled us to complete a series of time dependent asymptotic analyses upon the wild-type (under stress) and mutant cases, revealing nine and ten timescales respectively. We see mRNA transcription being dominant on the early timescales, with protein translation closely following for those mRNAs. As protein levels increase, inhibition of relevant transcription begins, decreasing certain mRNA concentrations. Finally, degradation comes into effect bringing all variables to steady state. By doing this process, we have broken down our nondimensional model (which does not have a full set of analytical solutions) into a step by step model of each dominant process. Thus, we are left with simplified models of our system, only taking into effect the dominant behaviours that control the GRN.

By performing this asymptotic analysis, we have also achieved asymptotic approximations to the steady states of the system, which were not analytically solvable in our full model. On most timescales we have full analytical solutions for each variable’s behaviour, enabling us to see the full breakdown of how each variable acts and how step by step the system evolves over time. By performing parameter variations upon the steady state values, we have been able to identify certain parameter groupings that have the most effect on the expression of efflux genes in both the wild-type and mutant case. For both cases, it was shown that both $\eta$ and $\xi$ relating to the binding affinity of EnvR to the two efflux pump genes and *envR* expression exhibited some of the strongest sensitivities. Thus showing evidence for the gene *envR* to be a potential inhibition target. This is biologically plausible as *envR* is the local repressor of the efflux gene *acrEF* but also can repress the gene *acrAB*. As this gene affects both pumps directly, by targeting *envR* we may be able to maximise inhibition of both efflux pumps. Notably, however, the processes of EnvR repression on the efflux pump genes were only dominant on the later timescales. On early timescales these repression processes did not appear at leading order. This may show the limitations of *envR* as an inhibition target, with the gene more likely to affect the long term behaviour only. In addition, more work needs to be done to consider the exact mechanisms by which EnvR interacts with the efflux genes, and we leave this for future work. Most other parameters exhibited a reasonable relative sensitivity, providing evidence that multiple genes could provide realistic inhibitory targets. Perhaps more importantly however, was the sensitivity of $\omega$ relating to the link between the concentration of AcrAB and the activation / repression of *acrEF*. Notably, this link appeared as a dominant process in both strains on the latter timescales. Whilst we do not currently know the full biological details of this link, the sensitivity of this parameter grouping suggests that it could provide a possible efflux inhibition target. This provides a strong case to delve into and further understand the mechanisms linking the various efflux pumps, as they could provide the key to inhibiting efflux.

Whilst delving into the steady state analysis has provided plentiful insights into efflux inhibition targets at the system’s long term behaviour, it is important to note that this does not fully encompass the system’s earlier behaviour. By summarising the asymptotic analysis showing the dominant behaviour on all timescales, we are able to exhibit a step by step breakdown of the system. With this summary, we were easier able to distinguish the differences of behaviour between the wild-type and mutant cases. In particular we noted the importance of *ramA* in the mutant case, with direct and indirect activation of *acrAB* through RamA. Whilst at steady state the indirect activation (via AcrR) is still prevalent, at leading order in the mathematical analysis the direct activation is not and appears to be an important factor in early activation of *acrAB* expression. This has given us reasonable grounds to consider *ramA* as a potential inhibition target, targeting *acrAB* expression directly at early time, and indirectly at long time. Although this gene may not be one of the most sensitive targets at long term behaviour, the step by step breakdown shows that the early interactions of the gene are of huge significance. Thus by targeting *ramA*, we may provide a method for inhibiting early efflux expression enough so that an antibiotic can kill the bacteria before its efflux pumps become fully active. The analysis has therefore also revealed the possible importance of effective timing of efflux inhibition and how this may vary between targets.

There are plenty of future steps that we could take with this model, the most obvious being including the dynamics of an antibiotic inducer. Whilst our current model gives us insight into how the genes interplay starting from a down-regulated state, it does not consider how an antibiotic concentration could itself affect the network. It is likely that in addition to antibiotic being expelled via the expressed efflux pumps, the presence of antibiotic will affect expression of certain areas of the network, hence creating a feedback loop. Other areas to explore are including stochastic events in the model. This could be incorporated within multiple processes in the model, for example: gene expression, degradation and binding dynamics. Finally, we could upscale the model to explore the effects of efflux expression on population growth and survival. This could be dependent on different environmental stressors and conditions. We believe that this work has provided useful insights into this GRN. With the hypotheses we have generated on potential inhibitory targets and pathways, this should provide evidence for further investigation of certain areas of the network and also to inspire potential therapies to be tested experimentally in order to combat efflux related MDR.
